# Quantitative ^1^H Nuclear Magnetic Resonance Assay for the Rapid Detection of Pyrazinamide Resistance in Mycobacterium tuberculosis from Sputum Samples

**DOI:** 10.1128/jcm.01522-22

**Published:** 2023-04-18

**Authors:** Juan M. Lopez, Mirko Zimic, Katherine Vallejos, Diego Sevilla, Mariella Quispe-Carbajal, Elisa Roncal, Joseline Rodríguez, Jhojailith Rodríguez, Ricardo Antiparra, Héctor Arteaga, Robert H. Gilman, Helena Maruenda, Patricia Sheen

**Affiliations:** a Departamento de Ciencias—Química, CERMN, Pontificia Universidad Católica del Perú, Lima, Perú; b Laboratorios de Investigación y Desarrollo, Facultad de Ciencias y Filosofía, Universidad Peruana Cayetano Heredia, Lima, Perú; c Johns Hopkins Bloomberg School of Public Health, Baltimore, Maryland, USA; University of Manitoba

**Keywords:** tuberculosis, pyrazinamide, drug susceptibility test, clinical trial, nuclear magnetic resonance, *Mycobacterium tuberculosis*, antibiotic resistance

## Abstract

Tuberculosis (TB), caused by Mycobacterium tuberculosis, is one of the 10 leading killer diseases in the world. At least one-quarter of the population has been infected, and there are 1.3 million deaths annually. The emergence of multidrug-resistant (MDR) and extensively drug-resistant (XDR) strains challenges TB treatments. One of the drugs widely used in first- and second-line regimens is pyrazinamide (PZA). Statistically, 50% of MDR and 90% of XDR clinical strains are resistant to PZA, and recent studies have shown that its use in patients with PZA-resistant strains is associated with higher mortality rates. Therefore, the is an urgent need for the development of an accurate and efficient PZA susceptibility assay. PZA crosses the M. tuberculosis membrane and is hydrolyzed to its active form, pyrazinoic acid (POA), by a nicotinamidase encoded by the *pncA* gene. Up to 99% of clinical PZA-resistant strains have mutations in this gene, suggesting that this is the most likely mechanism of resistance. However, not all *pncA* mutations confer PZA resistance, only the ones that lead to limited POA production. Therefore, susceptibility to PZA may be addressed simply by its ability to form, or not, POA. Here, we present a nuclear magnetic resonance method to accurately quantify POA directly in the supernatant of sputum cultures collected from TB patients. The ability of the clinical sputum culture to hydrolyze PZA was determined, and the results were correlated with the results of other biochemical and molecular PZA drug susceptibility assays. The excellent sensitivity and specificity values attained suggest that this method could become the new gold standard for the determination of PZA susceptibility.

## INTRODUCTION

Pyrazinamide (PZA) is an antituberculosis prodrug recommended by the WHO, which is widely used in first- and second-line regimens (1). Contrary to common antibiotics, which are normally active against growing bacteria and have little activity against persister mycobacteria, PZA has little activity against growing Mycobacterium tuberculosis and is active mainly against persister cells (2). The use of PZA has been shown to help reduce the duration of treatment to ~6 months and prevent relapse.

The molecular mechanism of PZA is not fully understood, although it has been prescribed since the 1950s (3–6). It is commonly accepted that PZA crosses the membrane of M. tuberculosis by passive diffusion; it is hydrolyzed to its active form, pyrazinoic acid (POA), in the cytosol (7) by pyrazinamidase (PZase), a nicotinamidase encoded by the *pncA* gene in M. tuberculosis (8), and POA is then expelled into the environment by an efflux system (9). In acidic medium (pH 5.5), POA is protonated, reenters the mycobacterium, and releases hydronium into the cytosol (9). The repetition of this cycle results in the accumulation of intracellular POA and the acidification of the cytoplasm, which is lethal for M. tuberculosis (9). The acidic pH not only allows POA to reenter and accumulate in the bacteria (9) but also lowers the membrane potential, inhibits the growth of the mycobacterium, and slows its metabolism. At neutral pH, the concentration of the anionic form of POA is higher, and it accumulates outside the cell. This explains why, *in vitro*, PZA is more active in acidic (pH 5.5) (10) than in neutral (pH 7 to 7.5) media (10, 11). Indeed, *in vitro*, PZA is active in cultures with bacteria under stress conditions, including starvation, acid pH, hypoxia, UV irradiation, and energy metabolism inhibition, among others (12–16). A correlation between PZA drug activity and decreasing M. tuberculosis metabolic activity has been suggested (7).

Up to 99% of clinical PZA-resistant strains have mutations in the *pncA* gene, suggesting that these mutations are the most likely mechanism of resistance to PZA (17, 18). Several mutations scattered along the *pncA* structural gene or the putative promoter region have been identified. These are mostly missense mutations, but some insertions, deletions, or nonsense mutations have also been reported. Moreover, K. Stoffels et al. demonstrated *in vitro* that mutations leading to PZA resistance occur at a high frequency, close to 10^−5^ (19). However, not all *pncA* mutations confer PZA resistance; only the ones that diminish PZase enzymatic activity or its expression and limit POA production lead to resistance (20, 21).

The use of PZA in patients infected with PZA-resistant M. tuberculosis strains is associated with higher mortality rates (22). Hence, its indiscriminate use in the growing number of patients with drug-resistant TB has become a public health problem. The situation is further complicated because there are no reliable microbiological drug susceptibility tests (DSTs) that could be used for routine analysis (1). The technical challenges in this area are numerous, including the influence of the inoculum size, the culture pH, the long turnaround time, and poor reproducibility (23, 24).

One of the most common DSTs is the mycobacterial growth indicator tube (MGIT) method, which is quite accurate for the detection of resistance to rifampicin (RIF) and isoniazid (INH) but not PZA (25). The commercial PZA-MGIT kit uses not only acidic culture medium (pH 6), affecting the growth rate, but also a PZA concentration (100 μg/mL) lower that the required critical value at this pH (26). The turnaround time for this indirect method, which requires the isolation and further incubation of M. tuberculosis (i.e., 26 to 55 days for culture detection in an Lowenstein Jenses (LJ) tube Becton Dickinson (BD) [27] and 4 to 21 days for detection with PZA-MGIT [28]), is in the range of 30 to 76 days.

Shorter turnaround times (5 to 21 days) are achieved with the microscopic-observation drug susceptibility (MODS) assay, an inexpensive procedure that accurately detects M. tuberculosis (29) and first-line (rifampicin and isoniazid) (30) and second-line (amikacin, capreomycin, ciprofloxacin, cycloserine, ethambutol, ethionamide, kanamycin, moxifloxacin, ofloxacin, *p*-aminosalicylic acid, and streptomycin) drug susceptibility (31) directly from sputum samples. For PZA, we recently reported a study addressing PZA susceptibility in sputum MODS cultures using higher concentrations of PZA (400 and 800 μg/mL) at near-neutral pH (pH 6.8) (32). These improvements yielded better sensitivity (76.9% to 89.7% for 400 μg/mL PZA and 71.8% to 82.1% for 800 μg/mL PZA) and specificity (93.0% to 97.9% for PZA at 400 μg/mL and 95.8% to 98.6% for PZA at 800 μg/mL) values (33).

Sequencing of the *pncA* gene in search of mutations associated with PZA resistance is another available indirect method. It is more accurate than the classical DSTs, but because not all of the mutations equally affect the PZase activity (21), the sensitivity is variable, and the specificity is low (33, 34). Furthermore, this molecular assay is limited by its high cost, the absence of mutation hot spots, and the existence of a wide repertoire of *pncA* gene mutations (17).

Due to the high correlation between the loss of PZase activity and M. tuberculosis PZA resistance (35), the sensitivity or resistance of a strain to PZA may be addressed simply by its ability to form, or not, POA. The classical Wayne test is a visual biochemical colorimetric assay that detects POA produced by PZA-susceptible M. tuberculosis strains. The M. tuberculosis samples are inoculated onto an agar culture medium containing 100 μg/mL PZA at near-neutral pH (pH 6.4 to 6.8). POA is released into the medium and detected by its reaction with ferrous ammonium sulfate (FAS) through the appearance of pink coloration. The absence of a color change indicates no POA formation and, therefore, PZA resistance (36). In order to reduce the turnaround time of this procedure, in 2019, we combined MODS culture with Wayne staining to detect POA directly in the supernatant of the sputum culture (MODS-Wayne) (37). The sputum samples are incubated until positive growth is detected by microscopy. PZA is then added at a concentration of 800 μg/mL. After 3 days of culture growth, POA is assessed through the pink coloration that develops after the addition of 10% FAS (37). However, due to matrix effects and protein precipitation, it does not allow accurate POA quantification by absorbance spectroscopy, and only qualitative observations can be performed (37). Hence, there is still no efficient and accurate PZA susceptibility assay for routine analysis in a clinic.

Nuclear magnetic resonance (NMR) is a highly accurate and reproducible analytical tool that allows the direct quantification of analytes in complex mixtures with minimal sample manipulation. This technique, widely used in metabolomics (38, 39), has also been employed in drug susceptibility assays (40, 41). Here, we present a novel method to quickly and accurately quantify POA and PZA in the supernatant of sputum cultures using NMR spectroscopy (MODS-NMR). This method allowed not only the monitoring of the kinetics of the transformation of PZA to POA but also the determination of a direct correlation between the amount of POA and PZA susceptibility. The results were compared to those of MODS-Wayne and a composite reference standard (CRS) test (1), confirmed by the results of PZA-MGIT, *pncA* gene sequencing (*pncA*-Seq), and the regular Wayne test. Finally, to have an indication of the effect of the bacterial concentration on the NMR results, the data were compared to bacilloscopic (BK) and MODS growth index (MODS-GI) data.

## MATERIALS AND METHODS

### TB sputum samples.

A total of 139 remnants from sputum clinical samples were collected from recently diagnosed and in-treatment patients from the Hospital Hipólito Unánue, Lima, Perú, and the Regional Tuberculosis Reference Laboratory, Callao, Lima, Perú. Ethical approval was obtained from the Universidad Peruana Cayetano Heredia (Constancia 345-15-19). Knowing that clinical multidrug-resistant (MDR) samples have an ~50% probability of also being resistant to PZA (42), for this study, a large number of samples resistant to rifampicin and/or isoniazid were selected.

### Bacilloscopy.

The acid-fast smear test was used to estimate the bacterial concentration in the sputum samples using Ziehl-Neelsen staining. The samples were classified as having a score of 0 (negative), 1+, 2+, or 3+ according to standard guidelines (43).

### MODS assay for MDR-TB detection (rifampicin and isoniazid).

Sputum samples were decontaminated according to a standardized protocol (43). Briefly, the sputum sample (2 mL) was mixed with an equal volume of an aqueous solution composed of *N*-acetyl-l-cysteine (0.5%), NaOH (2%), and sodium citrate (1.5%) and vortexed thoroughly. The mixture was incubated for 15 min at room temperature and centrifuged at 3,000 × *g* for 15 min at 17°C. The pellet was then resuspended in 2.5 mL of phosphate-buffered saline. For the culture assay, 500 μL of the decontaminated suspension was added to 7 mL of Middlebrook 7H9 medium containing 0.31% glycerol, 1.25 g of Bacto Casitone per L, 10% OADC (oleic acid, albumin, dextrose, and catalase), and 160 μL of PANTA. The culture assays were performed on a 24-well plate. For the control samples (2 wells), 900 μL of the diluted decontaminated sample was mixed with 100 μL of the culture medium. In the case of the isoniazid and rifampicin assays, 900 μL of the diluted decontaminated sample was mixed with 100 μL of 4 μg/mL of INH (1 well) and 1 μg/mL of RIF (1 well), respectively. The samples were incubated at 37°C for up to 21 days. M. tuberculosis strains H37Rv and DM97 were used as drug-sensitive and drug-resistant controls, respectively, at a final concentration of 2 × 10^5^ CFU/mL. The cordon-pattern bacterial growth in the control wells was observed using an inverted microscope (TS100; Nikon) at a ×40 magnification from day 4 of incubation, according to the following MODS-GI scale: index 1 is growth covering 25% of the well area, index 2 is growth covering 25 to 50% of the well area, index 3 is growth covering 50 to 75% of the well area, and index 4 is growth covering 100% of the well area (see Fig. S1 in the supplemental material). Susceptibility to INH and RIF was reported when cordon-pattern growth was observed in the INH- and RIF-containing wells (44).

### MODS-Wayne.

Drug susceptibility to PZA was evaluated according to the MODS-Wayne protocol (37) in a P3 laboratory. Briefly, 900 μL of the diluted decontaminated sample prepared as described above was incubated until cordon-pattern growth was observed. The plate was further incubated for 6 days, and 100 μL of an aqueous PZA solution (8 mg/mL) was then added. The plate was incubated for three more days. Finally, 800 μL of this mixture was recovered and divided equally into two tubes. One tube (400 μL) was used for the MODS-Wayne test, and the other tube (400 μL) was reserved for the MODS-NMR analysis. The tubes were then heated at 100°C for 30 min to kill the bacteria. A 10% ferrous ammonium sulfate aqueous solution (40 μL) was added to the MODS-Wayne tube. Positive results (PZA sensitive) are indicated by the reddish coloration formed after the addition of FAS. The intensity of the color was classified as low (index 1), mid (index 2), high (index 3), and extreme (index 4). A negative result (PZA resistant) is determined by the complete absence of color (index 0) (Fig. S2).

### MODS-NMR.

A heat-treated sample containing 400 μL of the MODS culture at 800 μg/mL of PZA was centrifuged at 10,000 rpm for 10 min, and the supernatant was removed from the P3 laboratory and stored at −20°C until use. Defrosted samples were transferred into 3-mm NMR tubes and inserted into a 5-mm tube containing 300 μL of D_2_O and 3 mM TMSP for the lock and frequency references. ^1^H-NMR experiments were recorded at 25°C using a Bruker Avance III HD 500-MHz spectrometer equipped with a TCI cryoprobe, an autosampler, and automatic tuning and matching. All spectra were acquired with a 30° flip angle, water presaturation at a 75-Hz field strength, 128 scans, 64,000 data points, a spectral width of 20 ppm, and a total relaxation time of 7.28 s. Spectra were Fourier transformed with 0.3-Hz exponential apodization and phase and baseline corrected using Topspin 3.6 software. The percentage of PZA transformed into POA was determined by integrating PZA signals at 9.02 ppm (*I*_1_), 8.65 ppm (*I*_2_), and 8.59 ppm (*I*_3_) and integrating POA signals at 8.91 ppm (*I*_1_) and 8.53 ppm (*I*_2_) (Fig. S3) in the ^1^H-NMR spectra according to the following equation:

$$\text{\% POA = }\frac{I_{\text{1}}^{\text{POA}}+I_{\text{2}}^{\text{POA}}}{I_{\text{1}}^{\text{POA}}+I_{\text{2}}^{\text{POA}}+I_{\text{1}}^{\text{PZA}}+I_{\text{2}}^{\text{PZA}}+I_{\text{3}}^{\text{PZA}}}$$

### Kinetics of POA production.

M. tuberculosis reference strains H37Rv, as a PZA-susceptible strain; DM97, as an MDR and PZA-resistant isolate; and H37Rv, as a PZA-resistant *pncA* knockout strain (45), were cultured in Middlebrook 7H10 medium containing OADC (7H10-OADC) for 14 days, and an inoculum comparable to a McFarland standard of 1 (3 × 10^8^ CFU/mL) was prepared. The inoculum was diluted 1:1,000 in 7H9-OADC broth to obtain 3 × 10^5^ CFU/mL, and 900 μL of this dilution was transferred to 8 wells of a 24-well plate. The plates were incubated at 37°C. Approximately 5 days after culture, when bacterial growth was observed using an inverted microscope (TS100; Nikon) at a ×40 magnification, 100 μL of 8 mg/mL of PZA was added to each of the wells. The samples were collected during a period of 7 days and tested by MODS-Wayne and MODS-NMR.

### Reference PZA susceptibility tests.

The MGIT test was performed according to the instructions provided by the manufacturer (28). The standard Wayne test was performed according to methods described previously by L. G. Wayne (36). The *pncA* sequencing test was performed according to methods described previously by Alcántara et al. (37).

## RESULTS

A total of 139 sputum samples from 112 patients were analyzed, of which 109 belonged to TB patients before drug treatment (day 0) and 26 belonged to patients after 7 days and 14 days of treatment. The full data are compiled in the supplemental material.

All of the samples were positive for tuberculosis by the MODS assay; 3.6% of the samples were found to be resistant to rifampicin, 22.3% were resistant to isoniazid, 47.5% were resistant to both drugs, and 26.6% were reported to be susceptible to both drugs.

In this study, the susceptibility of M. tuberculosis to PZA was assessed by three different assays: the mycobacterial growth indicator tube (MGIT) assay, the standard Wayne test, and *pncA* gene sequencing. Of the 139 samples analyzed, 35.3% were found to be resistant to PZA according to the MGIT assay, 24.5% were resistant according to the standard Wayne assay, and 30.9% were resistant according to *pncA* sequencing (see Table S1 in the supplemental material). *pncA* sequencing analysis reported 15 different mutations in 45 samples, 2 of which contained the G124A mutation, reported as susceptible; 39 of which contained mutations that were found to confer PZA resistance (Table S2); and 4 of which contained the F81S mutation, described as susceptible under the category of uncertain significance in the WHO catalogue of mutations (46). However, more recent studies have demonstrated that mutation F81S leads to resistance (17, 47–50). The complete list of resistant and susceptible mutations is detailed in Table S2. In summary, 23.0% were classified as PZA resistant by the three DSTs, 3.6% were resistant by the MGIT assay and *pncA*-Seq and susceptible by the standard Wayne assay, 4.3% were susceptible by the MGIT and Wayne assays and resistant by *pncA*-Seq, 8.6% were resistant by the MGIT assay and susceptible by the Wayne test and *pncA*-Seq, 1.4% were resistant by the Wayne test and susceptible by the MGIT assay and *pncA*-Seq, and 59.0% were found to be susceptible by the three tests. By applying the composite reference standard (CRS) rule (51), 26.6% of the samples (positive in two out of three component tests) are considered resistant to PZA (Table S3).

**FIG 1:**
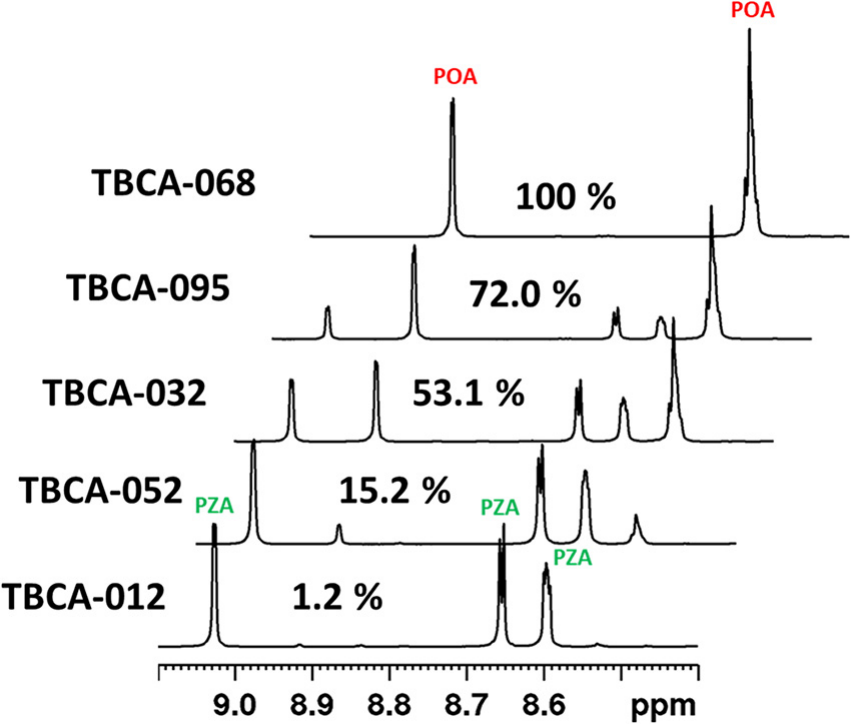
^1^H-NMR spectra of M. tuberculosis-positive MODS culture supernatants from the sputum samples of five different M. tuberculosis patients (samples TBCA-012, TBCA-032, TBCA-052, TBCA-068, and TBCA-95) after 3 days of incubation with PZA. The percentage of POA released was determined by integrating the PZA and POA signals.

Susceptibility to PZA was also evaluated directly in MODS cultures using the colorimetric qualitative MODS-Wayne assay and the quantitative proton NMR spectroscopy (^1^H-NMR) method. The MODS-Wayne test allowed the visual grouping of the samples according to the magnitude of the color change (indices 0 to 4), representative of the amount of POA formed (Fig. S2). In the case of the ^1^H-NMR method, implemented to determine the rate of hydrolysis PZA to POA by integrating the signals of the aromatic hydrogens of PZA at 9.02 ppm, 8.65 ppm, and 8.59 ppm and of POA at 8.91 ppm and 8.53 ppm (Fig. S3), the samples were grouped according to the percent POA value, a quantitative and accurate measurement. As shown in Fig. 1, the various rates of hydrolysis of PZA attained in this study are exemplified with sputum samples TBCA-012, TBCA-032, TBCA-052, TBCA-068, and TBCA-095.

**FIG 2:**
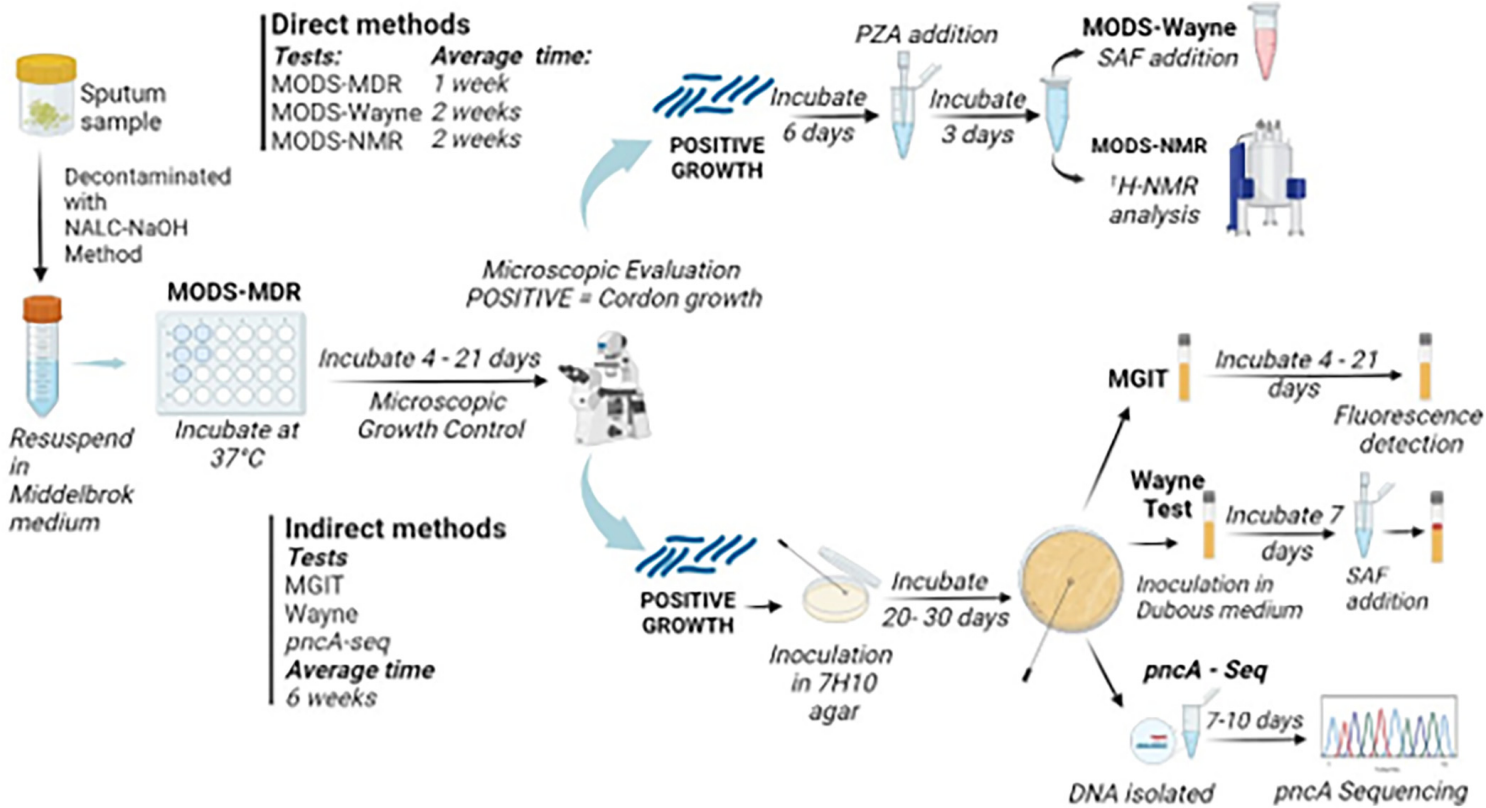
Timeline for PZA drug susceptibility assays. NALC, *N*-acetyl-l-cysteine. (Created with Biorender.com.)

One of the main advantages of the MODS-Wayne and MODS-NMR methods is that they are direct and fast (14 to 21 days) compared to indirect conventional tests such as the MGIT assay, the classic Wayne test, and *pncA*-Seq, methods which require the isolation of the mycobacteria prior to the analysis, extending the duration of the assay from 4 to 8 weeks (Fig. 2).

### PZA hydrolysis kinetics monitored by NMR.

**FIG 3:**
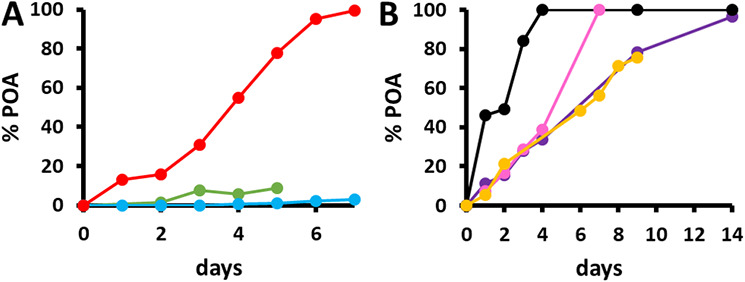
Kinetic curves of PZA conversion to POA monitored by ^1^H-NMR-MODS in three different M. tuberculosis strains. (A) H37Rv (PZA susceptible) (in red), DM97 (PZA resistant) (in green), and a *pncA* knockout strain (in light blue). (B) PZA kinetics of the following four clinical sputum samples, susceptible to PZA according to the MGIT assay, the Wayne test, and *pncA*-Seq (WT strains): TBN-155 (in pink), TBN-177 (in yellow), TBN-211 (in black), and TBN-212 (in violet).

NMR is an accurate and highly reproducible technique that allows the monitoring of the kinetics of the hydrolysis of PZA directly in the MODS culture supernatants. The ability of M. tuberculosis strains to hydrolyze PZA in the MODS cultures was first evaluated using H37Rv (PZA susceptible), DM97 containing PZA-resistant mutation T135P at the *pncA* gene level (20), and a *pncA* knockout strain. As expected, a significant difference in the concentrations of POA was observed among the samples (Fig. 3A). After 5 days, H37Rv processed 77.6% of the PZA and reached 100% hydrolysis in 7 days, whereas after 5 days, DM97 produced only 8.9% POA. These results show a clear correlation between the T135P mutation and the decreased ability to hydrolyze PZA. These results correlate well with those of our previous studies in which the PZase enzymatic activity of recombinant PZase harboring the T135P mutation was demonstrated to be lower (0.02 μmol POA min^−1^ mg^−1^) than that of wild-type (WT) PZase (38.4 μmol POA min^−1^ mg^−1^) (20).

The variability in the kinetic rates of four clinical sputum samples susceptible to PZA was also addressed (Fig. 3B). In 4 days, TBN-211 hydrolyzed 100% of the PZA, while in the case of TBN-212 and TBN-155, this value was reached at days 7 and 14, respectively. Sample TBN-177 reached 75.4% hydrolysis in 9 days. The kinetics of sample TBN-177 was also monitored by the MODS-Wayne semiquantitative assay, and the results, with a clear increase in coloration as a function of time (Fig. S4), correlate with the ^1^H-NMR kinetics data.

### MODS-NMR susceptibility assay.

In a previous study, using Wayne’s staining of MODS cultures, we determined that incubation with PZA for 3 days was sufficient to achieve good discrimination between susceptible and resistant strains (37). For comparison purposes, in this study, the same incubation time was used prior to the ^1^H-NMR reading.

**FIG 4:**
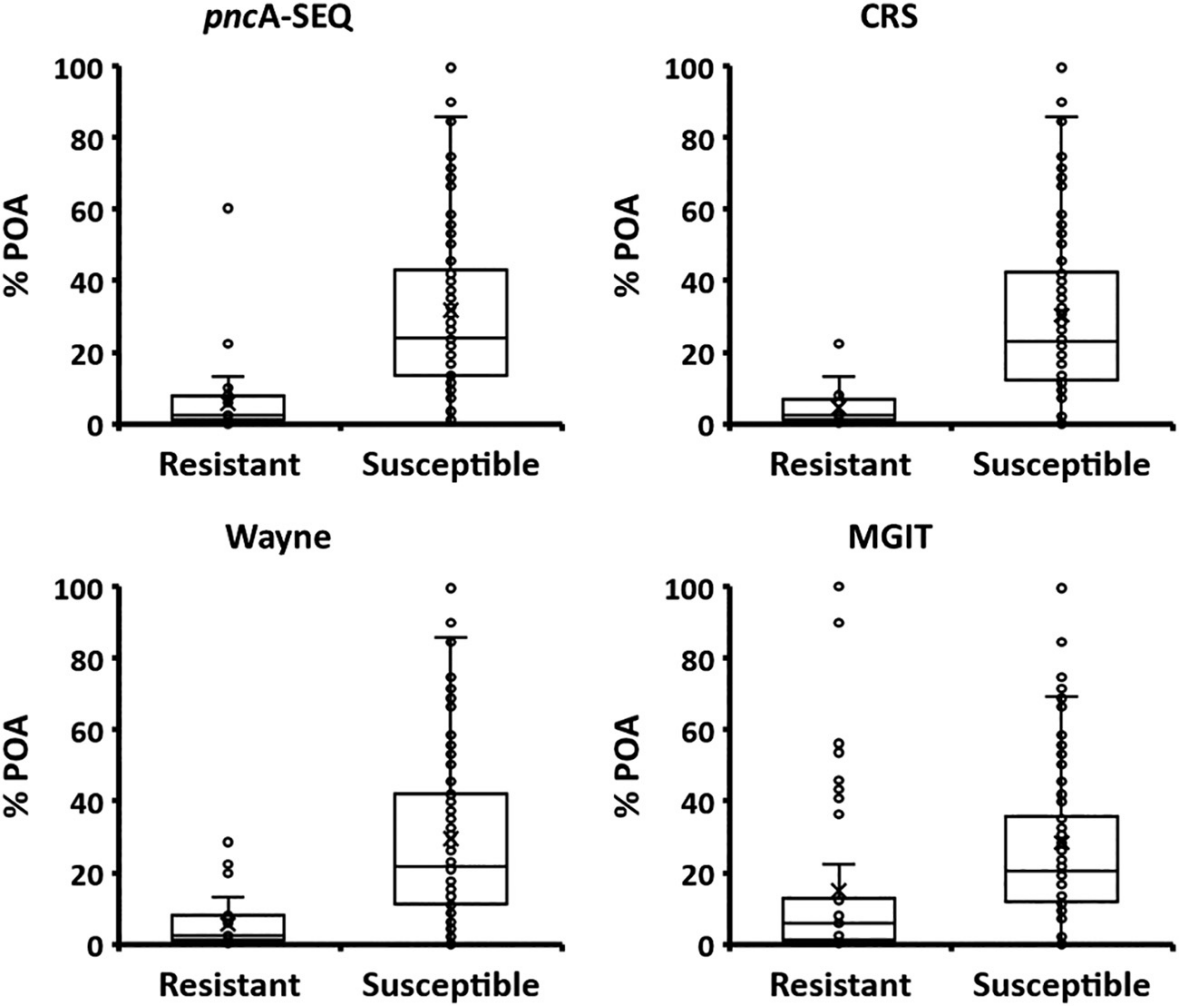
Percent PZA conversion to POA determined by ^1^H-NMR-MODS associated with PZA-resistant and PZA-susceptible M. tuberculosis strains classified according to *pncA* sequencing, the standard Wayne test, the MGIT assay, and the CRS test. Statistical values are reported in Table S4 in the supplemental material.

The percent conversion of PZA to POA was determined by NMR, and the values were grouped according to the type of strain, PZA resistant or PZA susceptible, based on the following classification tests performed as described above: the MGIT assay, the standard Wayne test, *pncA* sequencing, and the CRS test (Fig. 4).

The results indicate a partition between the two groups with respect to the percent POA values (Fig. 4) in all four DSTs: the MGIT assay (average of 28.4%, standard deviation of 23.4%, median of 20.4%, *Q*_1_ of 12.0%, and *Q*_3_ of 35.6% for susceptible strains; average of 15.0%, standard deviation of 23.2%, median of 5.9%, *Q*_1_ of 1.3%, and *Q*_3_ of 12.8% for resistant strains [*P* = 1.7 × 10^−3^]), the standard Wayne test (average of 29.5%, standard deviation of 24.5%, median of 21.7%, *Q*_1_ of 11.2%, and *Q*_3_ of 42.1% for susceptible strains; average of 5.6%, standard deviation of 6.6%, median of 2.4%, *Q*_1_ of 1.2%, and *Q*_3_ of 8.3% for resistant strains [*P* = 1.8 × 10^−7^]), *pncA* sequencing (average of 31.7%, standard deviation of 24.5%, median of 24.0%, *Q*_1_ of 13.4%, and *Q*_3_ of 43.1% for susceptible strains; average of 5.7%, standard deviation of 9.5%, median of 2.4%, *Q*_1_ of 1.1%, and *Q*_3_ of 7.9% for resistant strains [*P* = 5.4 × 10^−10^]), and the CRS test (average of 30.4%, standard deviation of 24.7%, median of 23.1%, *Q*_1_ of 12.3%, and *Q*_3_ of 42.2% for susceptible strains; average of 4.4%, standard deviation of 4.5%, median of 2.4%, *Q*_1_ of 1.1%, and *Q*_3_ of 7.0% for resistant strains [*P* = 5.6 × 10^−9^]) (for full data, see Table S4 in the supplemental material).

An important observation derived from the data is that a low percent POA value has a higher probability of assigning a resistant strain with less error and that a high percent POA value has a higher probability of correctly assigning a true-susceptible strain. Although there is a clear discrimination between the two groups, the numbers indicate that the range of percent POA values for susceptible strains is broader than that for resistant strains, with some extent of overlap. Better discrimination is obtained with respect to *pncA* sequencing, the standard Wayne test, and the CRS test than for the MGIT assay (Fig. 4 and Table S4) as the MGIT resistant group displays higher average (15.0%) and median (5.9%) percent POA values, and the *Q*_3_ MGIT resistant value (12.8%) is higher than the *Q*_1_ MGIT susceptible value (12.0%) (Table S4). In addition, for the resistant group, the MGIT data show 8 statistical outliers with high percent POA values, compared to only 3 in the standard Wayne test, 2 in the case of *pncA*-Seq, and 1 by CRS classification (Fig. 4).

**FIG 5:**
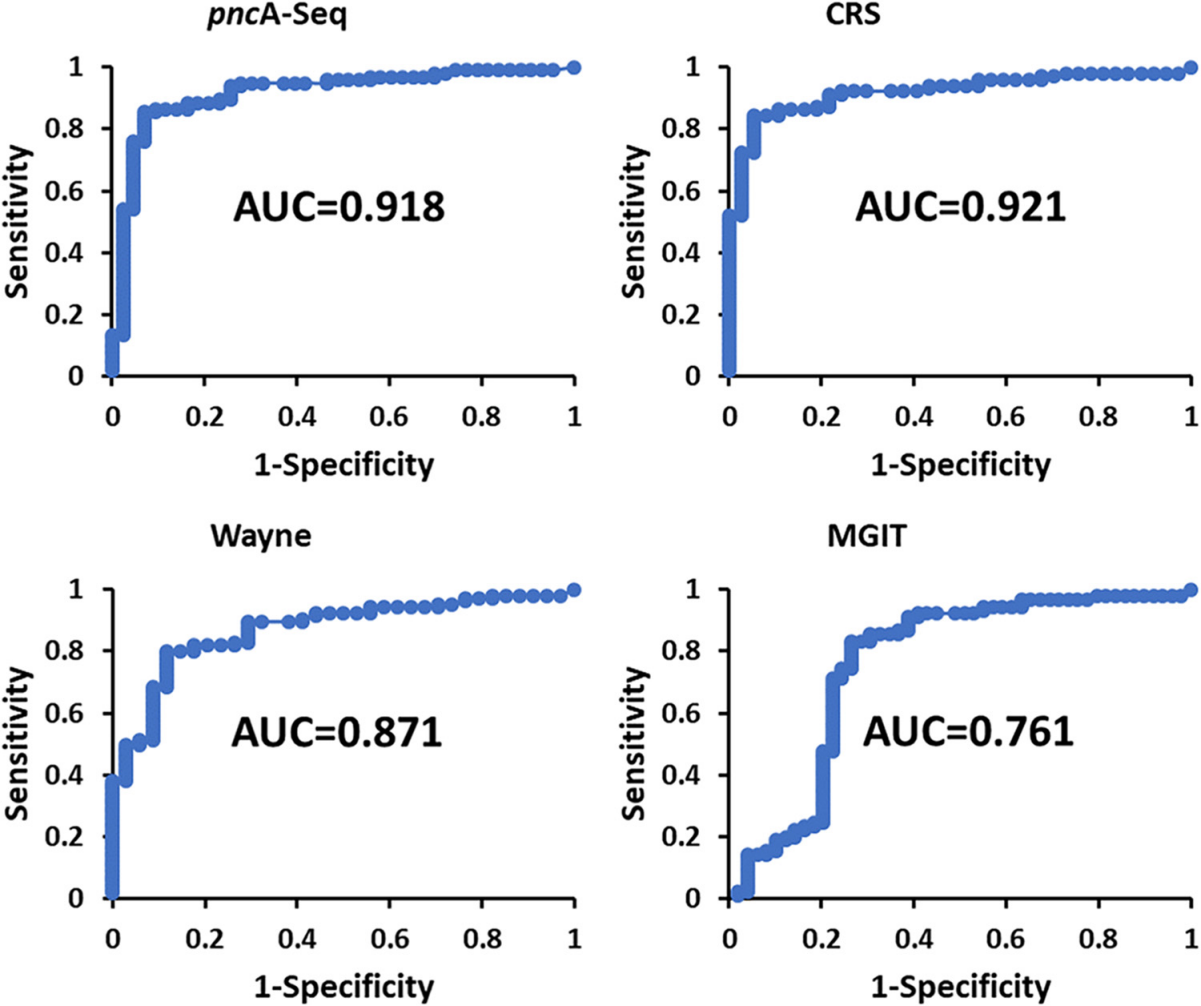
ROC curves for the percentage of PZA hydrolyzed to POA compared against classifications performed by the MGIT assay, the standard Wayne test, *pncA* sequencing, and the CRS test. AUC, area under the curve.

The NMR diagnostic thresholds were evaluated by receiver operating characteristic (ROC) curves. The quantitative NMR diagnostic model showed high predictability compared to *pncA* sequencing (area under the curve [AUC] = 0.918) and the standard Wayne test (AUC = 0.871) but much lower predictability than for the MGIT assay (AUC = 0.761) (Fig. 5). The agreement with the consensus CRS (AUC = 0.921) was better than that with the MGIT and standard Wayne tests alone and was very close to the values attained against the *pncA*-Seq data (Fig. 5). The optimal threshold for the percentage of POA by the NMR method was determined by the *G* mean. At 9.4% POA, high sensitivity and specificity values were achieved compared with data from the standard Wayne test (sensitivity = 80.0%, specificity= 88.2%, and kappa index = 0.584), *pncA* sequencing (sensitivity = 86.5%, specificity = 88.4%, and kappa index = 0.716), and the CRS test (sensitivity = 84.3%, specificity = 94.6%, and kappa index = 0.704). Once again, lower values were achieved when correlating the data against MGIT results (sensitivity = 83.3%, specificity = 73.5%, and kappa index = 0.563) (Table S5).

In addition, ROC plots display two interesting cutoff values. With a threshold set at 7.4% POA, the assay showed higher sensitivity and acceptable specificity for all four methods: the CRS test (sensitivity = 91.2%, specificity = 78.4%, and kappa index = 0.690), *pncA* sequencing (sensitivity = 93.8%, specificity = 74.4%, and kappa index = 0.704), the regular Wayne test (sensitivity = 86.7%, specificity = 70.6%, and kappa index = 0.551), and the MGIT assay (sensitivity = 91.1%, specificity = 61.2%, and kappa index = 0.552) (Fig. 5 and Table S5). On the other hand, a value of >20.6% POA reduced the sensitivity of the prediction but was highly specific (sensitivity = 52.0% and specificity = 100% for the CRS test, sensitivity = 54.2% and specificity = 97.7% for *pncA* sequencing, sensitivity = 49.5% and specificity = 97.1% for the Wayne test, and sensitivity = 47.8% and specificity = 79.6% for the MGIT assay) (Fig. 5 and Table S5).

In short, a value of <9.4% POA is associated with resistance to PZA, and a value of >9.4% is associated with susceptibility to PZA. With a percent POA value of <7.4% the probability of a false-negative result decreases substantially, and with a value of >20.6%, the probability of a false-positive result diminishes drastically.

### Evaluation of the mycobacterial concentration to assess the robustness of the NMR method.

**FIG 6:**
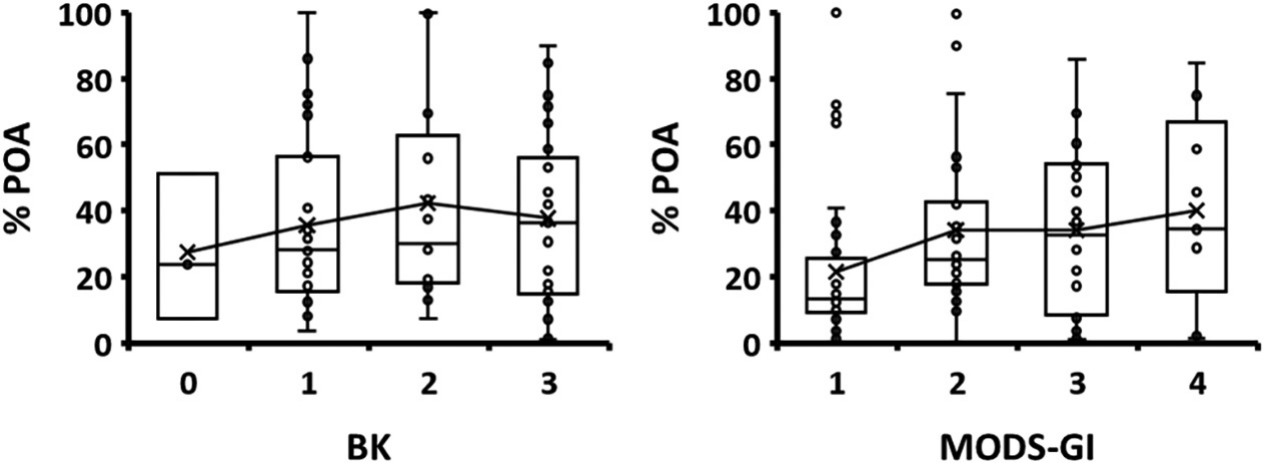
Percentage of PZA hydrolyzed to POA determined by ^1^H-NMR-MODS classified according to BK and MODS-GI. Only data from patients diagnosed as being positive by the CRS test were used in this analysis. The statistical data are reported in Tables S6 and S7 in the supplemental material.

It is clear that the rates of PZA conversion to POA are related to mutations in *pncA*, but the bacterial concentration could be playing a role as well. To evaluate this effect, the NMR results were compared to sputum bacilloscopy (BK) and MODS growth index data (Fig. 6). For this evaluation, only patients diagnosed as positive by the CRS test were considered. Statistical parameters are summarized in Tables S6 and S7.

The results indicate that the rate of formation of POA does not correlate with BK as no statistical difference among the BK groups was found by one-way analysis of variance (ANOVA) (Table S6). Since the measurement of BK is performed directly in the sputum of the patient, this lack of correlation was expected as PZA is added after a positive MODS observation is achieved.

**FIG 7:**
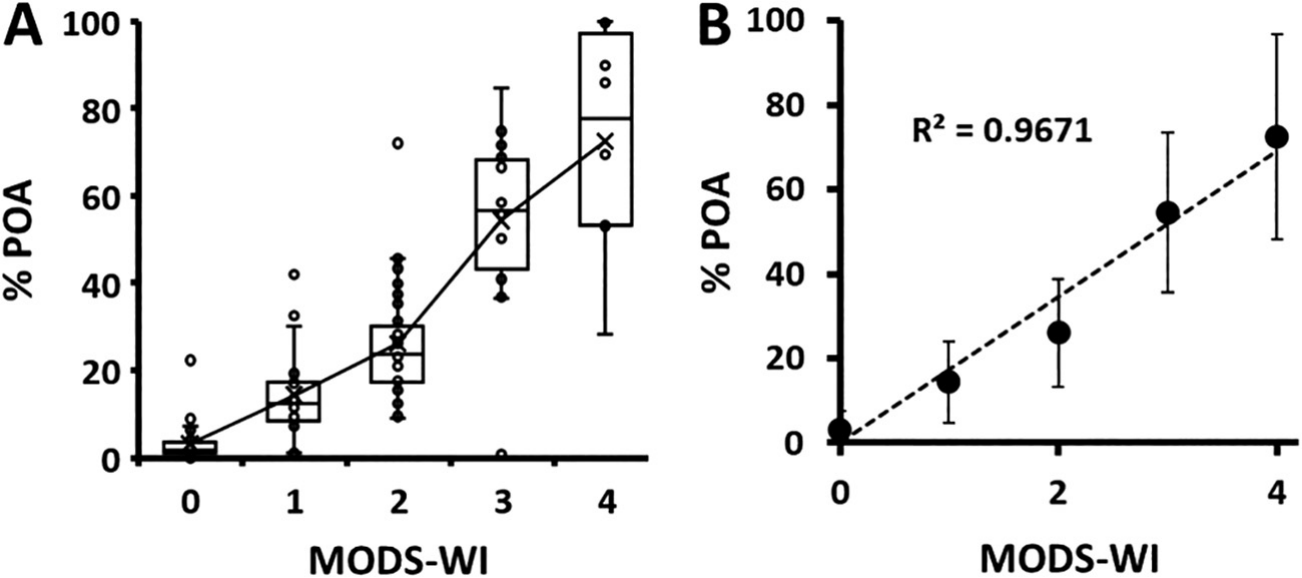
Percentage of PZA hydrolyzed to POA determined by ^1^H-NMR-MODS associated with MODS-WI groups. (A) Box plot for each MODS-WI group. The statistical values are reported in Table S9 in the supplemental material. (B) Average percentage of POA as a function of the MODS-WI. Bars represent the standard deviations.

In the case of MODS-GI, the estimation of the bacterial concentration was performed on the first day of cordon-pattern growth observation. One-way ANOVA of the full data revealed no statistical difference among the four index groups (*P* value of 0.099 by ANOVA). However, a significant difference was observed (*P* value of 3.9 × 10^−4^ by ANOVA) (Fig. 7 and Table S7) with the removal of the statistical outliers. Tukey’s *post hoc* HSD (honestly significant difference) test revealed that the average percentage of POA obtained with the samples belonging to MODS growth index 1 is noticeably lower than the others (Table S8). No major differences were found among indices 2, 3, and 4 (Table S8). Although the average value of index 1 is lower than the others (Fig. 6), this method seems to tolerate some variability in the initial concentration of mycobacteria.

### Validation of the MODS-Wayne test with MODS-NMR.

MODS-Wayne assays allow a visual qualitative assessment of POA by the color change that takes place after the addition of FAS. Even though a visual color scale related to the POA concentration from 0 to 4 (Fig. S2) is defined, the results are still subjective. Irrespective of this, the accurate quantification of PZA and POA by ^1^H-NMR, compared with the MODS Wayne index (MODS-WI), yielded a good linear correlation (Fig. 7). The statistical parameters are summarized in Table S9.

As shown in Table S5, the MODS-Wayne index 1 threshold showed higher sensitivity but lower specificity for the CRS (sensitivity = 92.0%, specificity = 80.0%, and kappa index = 0.713), *pncA*-Seq (sensitivity = 94.7%, specificity = 75.6%, and kappa index = 0.728), standard Wayne test (sensitivity = 88.4%, specificity = 75.0%, and kappa index = 0.607), and MGIT (sensitivity = 92.1%, specificity = 61.7%, and kappa index = 0.569) groups. These magnitudes relate better with the values obtained for a threshold of 7.4% POA (Table S5) than with those at the optimal 9.4% POA NMR cutoff.

The comparison of the distribution of percent POA NMR values against the color index data indicates that 7.4% POA is found within the lower limit of MODS-Wayne index 1 (average percent POA = 14.4%; standard deviation = 9.6%) and the upper limit of MODS-Wayne index 0 (average percent POA = 3.2%; standard deviation = 4.3%), suggesting that the human-eye threshold could be too low to correctly classify resistant strains based on a slight color change (Fig. 7 and Table S9).

### Assessment of diagnostic repeatability.

**FIG 8:**
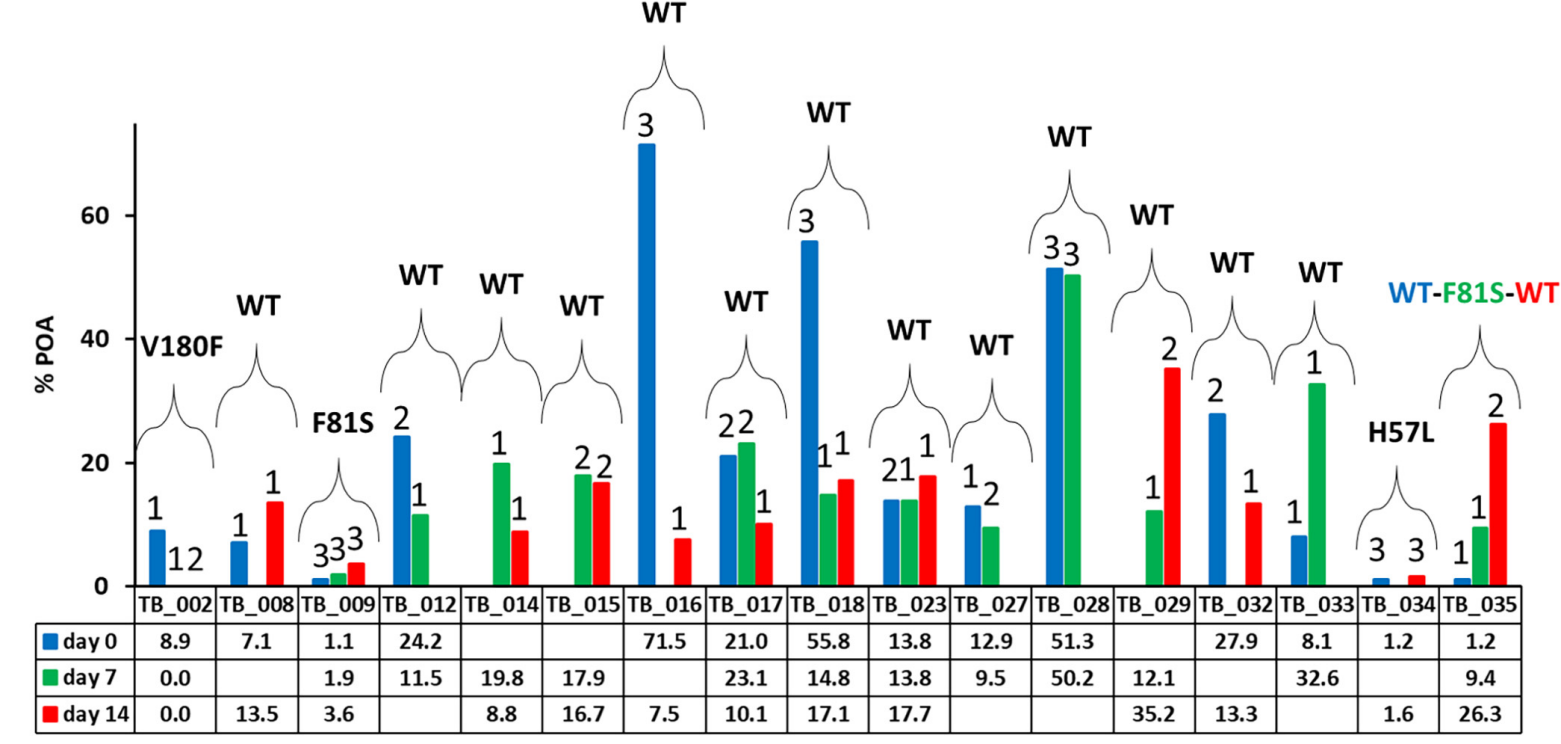
Percentage of PZA hydrolyzed to POA determined by ^1^H-NMR-MODS compared to the MODS-GI and *pncA* data for 17 patients. Sputum samples were collected on three different days of TB treatment (day 0, day 7, and day 14). In the table, the percent POA values are reported. Empty boxes correspond to samples that were not collected. All sample data are summarized in Table S10 in the supplemental material.

To assess diagnostic repeatability, different samples from the same patient were collected on different days of treatment: day 0, day 7, and/or day 14. A total of 40 samples were collected from 17 patients, and the data are shown in Table S10. Most of the results obtained for each patient were consistent across all tests performed. However, some differences were observed. First, for patients carrying susceptible strains according to the CRS test, TB_008, TB_014, TB_016, and TB_033, the MODS-NMR diagnostic results do not coincide (Fig. 8) for all samples. At least one of the values indicates resistance, and the other indicates susceptibility (Fig. 8). However, for all misclassified samples, the percent POA values were higher than 7%, and the MODS growth index was 1 (Fig. 8).

In the case of patient TB_002, carrying a resistant V180F strain, the discrepancies were at the level of the standard Wayne test, the MGIT assay, and MODS-Wayne. For day 0, the MODS-Wayne result (susceptible) does not agree with the results of all of the other tests, which indicates resistance. For day 14, the Wayne test (susceptible) and MGIT (susceptible) results do not agree with the MODS-Wayne result (resistant), nor do they agree with the values of the Wayne and MGIT tests reported for day 0 (resistant) and day 7 (resistant). These three samples, according to MODS-NMR, were classified as resistant (day 0, 8.9% POA; day 7, 0% POA; day 14, 0% POA), in agreement with the *pncA* sequencing classification (Fig. 8 and Table S10).

Similar inconsistencies were observed with the results gathered for patient TB_009. First, according to *pncA* sequencing, the M. tuberculosis strain is resistant (V180F mutation). The results of only two tests, MGIT on day 0 and MODS-Wayne on day 14, coincide with this result. In this case, again, the MODS-NMR results (percent POA values of between 1.1 and 3.6% at a <9.4% threshold) coincide with the sequencing data (Fig. 8 and Table S10).

Only one inconsistency in the *pncA* sequencing data was detected, and this occurred for a patient with an MDR rifampicin- and isoniazid-resistant strain, TB_035, showing the WT at day 0, F81S at day 7, and the WT at day 14. In this case, most of the results are inconsistent with each other, including the MODS-NMR results for day 14. The standard Wayne, MGIT, and MODS-Wayne assays indicate a susceptible strain for the three evaluated days, whereas the MODS-NMR results on day 0 and day 7 coincide with the MDR data (Fig. 8 and Table S10).

### Analysis of discordant results.

Currently, PZA drug susceptibility assays, whether genetic or phenotypic, display limitations that render them inappropriate as gold-standard methods. Therefore, some discrepancy across the DST data obtained was expected, and this was evaluated by the measurement of kappa coefficients of agreement. Indirect phenotypic methods such as the standard Wayne and MGIT assays show better correlations between them (kappa index of 0.678 for the Wayne test versus MGIT) than with the direct phenotypic MODS-NMR and MODS-Wayne assays (kappa indices of 0.584 for the Wayne test versus MODS-NMR, 0.607 for the Wayne test versus MODS-Wayne, 0.563 for the MGIT assay versus MODS-NMR, and 0.569 for the MGIT assay versus MODS-Wayne). Similarly, good agreement exists between the two direct phenotypic methods MODS-NMR and MODS-Wayne (kappa index of 0.711 for MODS-NMR versus MODS-Wayne) (Table S11). In the case of the indirect genotypic method *pncA*-Seq, the analysis showed high agreement with all phenotypic DST results (kappa indices of 0.768 for *pncA*-Seq versus the Wayne test, 0.708 for *pncA*-Seq versus the MGIT assay, 0.7167 for *pncA*-Seq versus MODS-NMR, and 0.728 for *pncA*-Seq versus MODS-Wayne) (Table S11).

The comparison of the results obtained from the analyses of the 139 sputum samples indicates that 29.5% of the samples display at least one discordant result. The full data for these 41 samples is shown in Table S12; 30 contain one discrepancy across the five methods, and 11 present more than one disagreement. The summarized results displayed in Tables S13 and S14 correlate well with the results of the analysis of kappa coefficients of agreement.

## DISCUSSION

The NMR method presented here accurately quantifies the hydrolysis of PZA to its active form POA in the supernatant of MODS cultures from clinical sputum samples. A clear relationship between the concentration of POA in the supernatant and the susceptibility of the MODS cultures to PZA was observed. Furthermore, ROC analyses of the NMR data allowed us to define thresholds for PZA susceptibility based on the percent POA concentration. A threshold of 9.4% POA, associated with higher sensitivity and higher specificity values than with the other methods (MGIT assay, Wayne test, *pncA*-Seq, and CRS test), was set as the optimal threshold. With a percent POA value of <7.4%, the probability of misdiagnosing a patient with a resistant strain (false negative) decreases considerably, and with a value of >20.6%, the probability of misdiagnosing a patient with a susceptible strain (false positive) also diminishes. Although there is clear discrimination between the PZA-resistant and PZA-susceptible groups, the range of percent POA values for susceptible strains is broader and overlaps to some extent the range of percent POA values for resistant strains. To further optimize this NMR drug susceptibility assay, the time of incubation with the drug and the initial concentration of PZA are variables that could be optimized. Probably, by lengthening the incubation time, the differences in percent POA values between susceptible and resistant strains will increase, raising the sensitivity and specificity of the method. These points deserve further analysis in future studies.

Since there is no reference method to accurately assess PZA susceptibility, the analyses of the NMR results presented here depend on the data obtained from other DSTs (MGIT assay, Wayne test, *pncA*-Seq, CRS test, and MODS-Wayne), all of which experience limitations.

First, the MGIT assay, which is based on M. tuberculosis growth, displays low sensitivity, specificity, and reproducibility as PZA requires acidic culture medium to be active, and such culture medium, by itself, inhibits M. tuberculosis growth. The MGIT drug susceptibility assay displays the highest rates of discrepancies with *pncA*-Seq, 12.2% (17 discrepancies); MODS-Wayne, 17.3% (24 discrepancies); and MODS-NMR, 19.4% (27 discrepancies), but a lower rate of discrepancies with the standard Wayne method, 13.7% (19 discrepancies) (see Table S14 in the supplemental material). Among these samples, we can highlight 11 of them that were classified as resistant by the MGIT assay while the rest of the DSTs classified them as susceptible (Tables S12 and S13).

Second, even though *pncA*-Seq has been demonstrated to be the most accurate of the classical DSTs, not all of the data associated with the mutations observed are readily available in the literature, and some discrepancies exist; in addition, not all *pncA* mutations imply a complete abolition of the enzymatic activity, only a decrease with the concomitant diminishment of the kinetics of POA formation. This becomes clear when comparing the NMR kinetics data between the DM97 PZA-resistant strain and the H37Rv PZA-susceptible strain; both produce POA, but the kinetics of DM97 is significantly slower (Fig. 3). Another limitation of DNA sequencing drug susceptibility determination is that, typically, a single colony is sequenced, raising the question of sample heterogeneity. This scenario was encountered with the data reported for patient TB_035 carrying an MDR (rifampicin- and isoniazid-resistant) strain. Two different strains were observed, the WT and an F81S mutant (PZA resistant), in the *pncA*-Seq analysis of the sputum samples collected from this patient on days 0, 7, and 14. These results bring up several issues that are currently under discussion: Can a patient be infected with more than one strain? How frequent is this event? In the case of coinfection with a resistant strain and a susceptible strain, what effect will the POA produced by the susceptible M. tuberculosis strain have on the development of the resistant strain? Finally, how does M. tuberculosis strain heterogeneity affect the results of DSTs? These points deserve further examination.

Third, the subjective nature of the regular Wayne and MODS-Wayne thresholds is a limitation of these DSTs. The analyses of PZA-resistant strains that produce small amounts of POA, for example, by the regular Wayne and MODS-Wayne tests could induce false-positive results because of the subtle color change produced by even small amounts of POA. This accounts for the rates of disagreement observed between genotypic *pncA*-Seq and the regular Wayne test (8.6%) and MODS-Wayne (10.1%) (Table S14). Most of the discrepancies correspond to samples with index values of 0 and 1. Five *pncA*-WT mycobacterial samples were found to be resistant according to MODS-Wayne (index of 0) (Tables S12 and S13). Eight samples turned susceptible (index of ≥1) by MODS-Wayne but were resistant according to *pncA*-Seq (L4S, sample TBCA-150; D49N, ME-27 and MP-505; F81S, TBN-131, TBN-153, and TBN-304; V180F, TBN-45; promotor mutation A-11G, MP-465). In the case of the standard Wayne test, only 2 cases involving *pncA*-WT strains were found to be resistant (Tables S12 and S13), and 10 samples carrying a resistance mutation (V9G, TBCA-171; Q10R, TBCA-174 and TBCA-214; H51R, TBCA-240 and TBCA-250; F81S, TBN-131, TBN-153, TBN-166, and TBN-304) were classified as susceptible strains. In both cases, the misclassification is probably connected to the subjectiveness involved in assessing mild color changes among the samples.

In contrast, NMR produces irrefutable quantitative values through which more reliable thresholds could be proposed to better discriminate PZA-susceptible from PZA-resistant strains. However, since there is no adequate reference method to compare the data against it, it is difficult to establish the threshold correctly. Even the optimal threshold of 9.4% POA determined by comparison with the CRS method displayed inconsistencies. Seven samples with POA values of <9.4% considered resistant by the MODS-NMR test were classified as susceptible strains according to the other methods (Table S13). However, in six of these cases, the percent POA values were quite close to the established threshold (TBN-90, 7.9% POA; TBN-120, 7.1% POA; TBN-188, 7.4% POA; TBN-194, 7.5% POA; TBN-202, 7.5% POA; TBN-231, 8.1% POA), which suggests the possibility of further refining the limit established here. Also, all of these samples yielded an index of 1 according to MODS-Wayne (Table S12), confirming their borderline nature. Similarly, the results for sample TBCA-215, also with a percent POA value somewhat close to the threshold, 13.3%, and also with a MODS index value of 1 (Table S12), therefore considered susceptible, did not agree with the resistance label indicated by the regular Wayne, MGIT, and *pncA*-Seq (mutation Q10R) methods. In addition to these cases, there is one case, sample TBN-297, in which a percent POA value clearly below the threshold level, 1.2%, was registered as susceptible by the CRS test. This discrepancy is well explained considering that this sample corresponds to patient TB_035, who was known to be carrying two different strains (WT and F81S) (Tables S10 and S12).

In addition, MODS-NMR was shown to be quite robust with respect to the M. tuberculosis concentrations in positive MODS cultures since differences in the MODS growth index or BK do not significantly affect the NMR susceptibility determination. However, standardization of the initial bacterial concentration with the MODS growth index or other means might lead to better results.

Another important point in the development of any tuberculosis susceptibility test is the turnaround time. Both MODS-NMR and MODS-Wayne allow results in almost 2 weeks (18.5 ± 5 days on average); however, the MGIT, regular Wayne, and *pncA*-Seq methods take much longer (4 to 8 weeks) because of the additional primary isolation step (20 to 30 days) (42).

In summary, an excellent correlation between the MODS-Wayne and MODS-NMR results was achieved, with an almost linear trend between the concentrations of POA and the qualitative Wayne color index data. Similarly, our MODS-NMR results were highly correlated with the *pncA*-Seq data. Furthermore, this new NMR method allows the inspection of the global effect of the mutation on the kinetics of POA formation and the direct correlation of this value with the susceptibility of the M. tuberculosis strain.

NMR analysis of clinical samples is not very common. However, its high sensitivity, its simplicity (well-defined nonoverlapping signals), and the highly deshielded aromatic resonances of PZA and POA (far from crowded signal regions associated with the metabolites present in the biological mixture) make this method particularly attractive to be implemented using a low-cost benchtop spectrometer amenable to hospital settings. The chemical shift difference between the two most deshielded signals from PZA (9.02 ppm; 2.7-Hz line width) and POA (8.91 ppm; line width of 2.5 Hz) is 0.11 ppm. This value corresponds to 8.8 Hz in an 80-MHz benchtop NMR spectrometer, which should provide enough resolution to separate the two peaks and provide accurate quantification.

We are convinced that the MODS-NMR method developed here will contribute to a better understanding of the PZA resistance mechanism and will become the new gold-standard DST for PZA susceptibility determination.

## References

[1] World Health Organization. 2021. Global tuberculosis report. World Health Organization, Geneva, Switzerland.

[2] Hu Y, Coates AR, Mitchison DA. 2006. Sterilising action of pyrazinamide in models of dormant and rifampicin-tolerant Mycobacterium tuberculosis. Int J Tuberc Lung Dis 10:317–322. 16562713

[3] Malome L, Schurr A, Lindh H, McKenzie D, Kiser JS, Williams JH. 1952. The effect of pyrazinamide (aldinamide) on experimental tuberculosis in mice. Am Rev Tuberc 65:511–518. 14924173

[4] Yeager RL, Munroe WG, Dessau FI. 1952. Pyrazinamide (aldinamide*) in the treatment of pulmonary tuberculosis. Trans Annu Meet Natl Tuberc Assoc 48:178–201. 13038888

[5] Fox W, Ellard GA, Mitchison DA. 1999. Studies on the treatment of tuberculosis undertaken by the British Medical Research Council tuberculosis units, 1946-1986, with relevant subsequent publications. Int J Tuberc Lung Dis 3:S231–S279. 10529902

[6] National Institute for Health and Care Excellence. 2016. NICE guideline. National Institute for Health and Care Excellence, London, United Kingdom.

[7] Zhang Y, Wade MM, Scorpio A, Zhang H, Sun Z. 2003. Mode of action of pyrazinamide: disruption of Mycobacterium tuberculosis membrane transport and energetics by pyrazinoic acid. J Antimicrob Chemother 52:790–795. doi:10.1093/jac/dkg446. 14563891

[8] Scorpio A, Zhang Y. 1996. Mutations in pncA, a gene encoding pyrazinamidase/nicotinamidase, cause resistance to the antituberculous drug pyrazinamide in tubercle bacillus. Nat Med 2:662–667. doi:10.1038/nm0696-662. 8640557

[9] Zhang Y, Scorpio A, Nikaido H, Sun Z. 1999. Role of acid pH and deficient efflux of pyrazinoic acid in unique susceptibility of Mycobacterium tuberculosis to pyrazinamide. J Bacteriol 181:2044–2049. doi:10.1128/JB.181.7.2044-2049.1999. 10094680 PMC93615

[10] McDermott W, Tompsett R. 1954. Activation of pyrazinamide and nicotinamide in acidic environments in vitro. Am Rev Tuberc 70:748–754. doi:10.1164/art.1954.70.4.748. 13197751

[11] Tarshis MS, Weed WA, Jr. 1953. Lack of significant in vitro sensitivity of Mycobacterium tuberculosis to pyrazinamide on three different solid media. Am Rev Tuberc 67:391–395. doi:10.1164/art.1953.67.3.391. 13031058

[12] Betts JC, Lukey PT, Robb LC, McAdam RA, Duncan K. 2002. Evaluation of a nutrient starvation model of Mycobacterium tuberculosis persistence by gene and protein expression profiling. Mol Microbiol 43:717–731. doi:10.1046/j.1365-2958.2002.02779.x. 11929527

[13] Wade MM, Zhang Y. 2004. Anaerobic incubation conditions enhance pyrazinamide activity against Mycobacterium tuberculosis. J Med Microbiol 53:769–773. doi:10.1099/jmm.0.45639-0. 15272064

[14] Wade MM, Zhang Y. 2006. Effects of weak acids, UV and proton motive force inhibitors on pyrazinamide activity against Mycobacterium tuberculosis in vitro. J Antimicrob Chemother 58:936–941. doi:10.1093/jac/dkl358. 16950824

[15] Huang Q, Chen Z-F, Li Y-Y, Zhang Y, Ren Y, Fu Z, Xu S-Q. 2007. Nutrient-starved incubation conditions enhance pyrazinamide activity against Mycobacterium tuberculosis. Chemotherapy 53:338–343. doi:10.1159/000107723. 17785970

[16] Gu P, Constantino L, Zhang Y. 2008. Enhancement of the antituberculosis activity of weak acids by inhibitors of energy metabolism but not by anaerobiosis suggests that weak acids act differently from the front-line tuberculosis drug pyrazinamide. J Med Microbiol 57:1129–1134. doi:10.1099/jmm.0.2008/000786-0. 18719183

[17] Yadon AN, Maharaj K, Adamson JH, Lai Y-P, Sacchettini JC, Ioerger TR, Rubin EJ, Pym AS. 2017. A comprehensive characterization of PncA polymorphisms that confer resistance to pyrazinamide. Nat Commun 8:588. doi:10.1038/s41467-017-00721-2. 28928454 PMC5605632

[18] Whitfield MG, Soeters HM, Warren RM, York T, Sampson SL, Streicher EM, van Helden PD, van Rie A. 2015. A global perspective on pyrazinamide resistance: systematic review and meta-analysis. PLoS One 10:e0133869. doi:10.1371/journal.pone.0133869. 26218737 PMC4517823

[19] Stoffels K, Mathys V, Fauville-Dufaux M, Wintjens R, Bifani P. 2012. Systematic analysis of pyrazinamide-resistant spontaneous mutants and clinical isolates of Mycobacterium tuberculosis. Antimicrob Agents Chemother 56:5186–5193. doi:10.1128/AAC.05385-11. 22825123 PMC3457413

[20] Sheen P, Ferrer P, Gilman RH, López-Llano J, Fuentes P, Valencia E, Zimic MJ. 2009. Effect of pyrazinamidase activity on pyrazinamide resistance in Mycobacterium tuberculosis. Tuberculosis (Edinb) 89:109–113. doi:10.1016/j.tube.2009.01.004. 19249243 PMC2691962

[21] Li K, Yang Z, Gu J, Luo M, Deng J, Chen Y. 2021. Characterization of pncA mutations and prediction of PZA resistance in Mycobacterium tuberculosis clinical isolates from Chongqing, China. Front Microbiol 11:594171. doi:10.3389/fmicb.2020.594171. 33505367 PMC7832174

[22] Collaborative Group for the Meta-Analysis of Individual Patient Data in MDR-TB treatment—2017, Ahmad N, Ahuja SD, Akkerman OW, Alffenaar J-WC, Anderson LF, Baghaei P, Bang D, Barry PM, Bastos ML, Behera D, Benedetti A, Bisson GP, Boeree MJ, Bonnet M, Brode SK, Brust JCM, Cai Y, Caumes E, Cegielski JP, Centis R, Chan P-C, Chan ED, Chang K-C, Charles M, Cirule A, Dalcolmo MP, D’Ambrosio L, de Vries G, Dheda K, Esmail A, Flood J, Fox GJ, Fréchet-Jachym M, Fregona G, Gayoso R, Gegia M, Gler MT, Gu S, Guglielmetti L, Holtz TH, Hughes J, Isaakidis P, Jarlsberg L, Kempker RR, Keshavjee S, Khan FA, Kipiani M, Koenig SP, Koh W-J, Kritski A, et al. 2018. Treatment correlates of successful outcomes in pulmonary multidrug-resistant tuberculosis: an individual patient data meta-analysis. Lancet 392:821–834. doi:10.1016/S0140-6736(18)31644-1. 30215381 PMC6463280

[23] Cui Z, Wang J, Lu J, Huang X, Zheng R, Hu Z. 2013. Evaluation of methods for testing the susceptibility of clinical Mycobacterium tuberculosis isolates to pyrazinamide. J Clin Microbiol 51:1374–1380. doi:10.1128/JCM.03197-12. 23390285 PMC3647927

[24] Zimic M, Loli S, Gilman RH, Gutierrez A, Fuentes P, Cotrina M, Kirwan D, Sheen P. 2012. A new approach for pyrazinamide susceptibility testing in Mycobacterium tuberculosis. Microb Drug Resist 18:372–375. doi:10.1089/mdr.2011.0207. 22372927 PMC3462410

[25] Tortoli E, Cichero P, Piersimoni C, Simonetti MT, Gesu G, Nista D. 1999. Use of BACTEC MGIT 960 for recovery of mycobacteria from clinical specimens: multicenter study. J Clin Microbiol 37:3578–3582. doi:10.1128/JCM.37.11.3578-3582.1999. 10523555 PMC85696

[26] Chedore P, Bertucci L, Wolfe J, Sharma M, Jamieson F. 2010. Potential for erroneous results indicating resistance when using the Bactec MGIT 960 system for testing susceptibility of Mycobacterium tuberculosis to pyrazinamide. J Clin Microbiol 48:300–301. doi:10.1128/JCM.01775-09. 19923479 PMC2812260

[27] Rageade F, Picot N, Blanc-Michaud A, Chatellier S, Mirande C, Fortin E, van Belkum A. 2014. Performance of solid and liquid culture media for the detection of Mycobacterium tuberculosis in clinical materials: meta-analysis of recent studies. Eur J Clin Microbiol Infect Dis 33:867–870. doi:10.1007/s10096-014-2105-z. 24760249

[28] Becton, Dickinson and Company. 2016. BACTEC MGIT 960 PZA kit for antimycobacterial susceptibility testing of Mycobacterium tuberculosis. Becton, Dickinson and Company, Sparks, MD.

[29] Kirwan DE, Ugarte-Gil C, Gilman RH, Rizvi H, Ticona E, Chavez G, Cabrera JL, Matos ED, Evans CA, Moore DAJ, Friedland JS, Lymph Node TB Working Group, Peru. 2016. Microscopic observation drug susceptibility assay for rapid diagnosis of lymph node tuberculosis and detection of drug resistance. J Clin Microbiol 54:185–189. doi:10.1128/JCM.02227-15. 26511739 PMC4702711

[30] Moore DAJ, Mendoza D, Gilman RH, Evans CAW, Hollm Delgado M-G, Guerra J, Caviedes L, Vargas D, Ticona E, Ortiz J, Soto G, Serpa J, Tuberculosis Working Group in Peru. 2004. Microscopic observation drug susceptibility assay, a rapid, reliable diagnostic test for multidrug-resistant tuberculosis suitable for use in resource-poor settings. J Clin Microbiol 42:4432–4437. doi:10.1128/JCM.42.10.4432-4437.2004. 15472289 PMC522377

[31] Fitzwater SP, Sechler GA, Jave O, Coronel J, Mendoza A, Gilman RH, Friedland JS, Moore DAJ. 2013. Second-line anti-tuberculosis drug concentrations for susceptibility testing in the MODS assay. Eur Respir J 41:1163–1171. doi:10.1183/09031936.00059812. 22903960

[32] Alcántara R, Fuentes P, Marin L, Kirwan DE, Gilman RH, Zimic M, Sheen P. 2020. Direct determination of pyrazinamide (PZA) susceptibility by sputum microscopic observation drug susceptibility (MODS) culture at neutral pH: the MODS-PZA assay. J Clin Microbiol 58:e01165-19. doi:10.1128/JCM.01165-19. 32132191 PMC7180241

[33] Bhuju S, Fonseca LDS, Marsico AG, de Oliveira Vieira GB, Sobral LF, Stehr M, Singh M, Saad MHF. 2013. Mycobacterium tuberculosis isolates from Rio de Janeiro reveal unusually low correlation between pyrazinamide resistance and mutations in the pncA gene. Infect Genet Evol 19:1–6. doi:10.1016/j.meegid.2013.06.008. 23770140

[34] Tan Y, Hu Z, Zhang T, Cai X, Kuang H, Liu Y, Chen J, Yang F, Zhang K, Tan S, Zhao Y. 2014. Role of pncA and rpsA gene sequencing in detection of pyrazinamide resistance in Mycobacterium tuberculosis isolates from southern China. J Clin Microbiol 52:291–297. doi:10.1128/JCM.01903-13. 24131688 PMC3911430

[35] Huang T-S, Lee SS-J, Tu H-Z, Huang W-K, Chen Y-S, Huang C-K, Wann S-R, Lin H-H, Liu Y-C. 2003. Correlation between pyrazinamide activity and pncA mutations in Mycobacterium tuberculosis isolates in Taiwan. Antimicrob Agents Chemother 47:3672–3673. doi:10.1128/AAC.47.11.3672-3673.2003. 14576145 PMC253789

[36] Wayne LG. 1974. Simple pyrazinamidase and urease tests for routine identification of mycobacteria. Am Rev Respir Dis 109:147–151. doi:10.1164/arrd.1974.109.1.147. 4203284

[37] Alcántara R, Fuentes P, Antiparra R, Santos M, Gilman RH, Kirwan DE, Zimic M, Sheen P. 2019. MODS-Wayne, a colorimetric adaptation of the microscopic-observation drug susceptibility (MODS) assay for detection of Mycobacterium tuberculosis pyrazinamide resistance from sputum samples. J Clin Microbiol 57:e01162-18. doi:10.1128/JCM.01162-18. 30429257 PMC6355525

[38] Markley JL, Brüschweiler R, Edison AS, Eghbalnia HR, Powers R, Raftery D, Wishart DS. 2017. The future of NMR-based metabolomics. Curr Opin Biotechnol 43:34–40. doi:10.1016/j.copbio.2016.08.001. 27580257 PMC5305426

[39] Emwas A-H, Roy R, McKay RT, Tenori L, Saccenti E, Gowda GAN, Raftery D, Alahmari F, Jaremko L, Jaremko M, Wishart DS. 2019. NMR spectroscopy for metabolomics research. Metabolites 9:123. doi:10.3390/metabo9070123. 31252628 PMC6680826

[40] García-Álvarez L, Busto JH, Avenoza A, Sáenz Y, Peregrina JM, Oteo JA. 2015. Proton nuclear magnetic resonance spectroscopy as a technique for gentamicin drug susceptibility studies with Escherichia coli ATCC 25922. J Clin Microbiol 53:2433–2438. doi:10.1128/JCM.00604-15. 25972417 PMC4508405

[41] García-Álvarez L, Busto H, Oteo JA. 2019. Proton nuclear magnetic resonance for antimicrobial drug susceptibility studies: why has progress been slow? Future Microbiol 14:1175–1177. doi:10.2217/fmb-2019-0216. 31625445

[42] Moore DAJ, Evans CAW, Gilman RH, Caviedes L, Coronel J, Vivar A, Sanchez E, Piñedo Y, Saravia JC, Salazar C, Oberhelman R, Hollm-Delgado M-G, LaChira D, Escombe AR, Friedland JS. 2006. Microscopic-observation drug-susceptibility assay for the diagnosis of TB. N Engl J Med 355:1539–1550. doi:10.1056/NEJMoa055524. 17035648 PMC1780278

[43] Stop TB Partnership. 2014. Mycobacteriology laboratory manual. Stop TB Partnership, Geneva, Switzerland.

[44] Calderón RI, Velásquez GE, Becerra MC, Zhang Z, Contreras CC, Yataco RM, Galea JT, Lecca LW, Kritski AL, Murray MB, Mitnick CD. 2017. Prevalence of pyrazinamide resistance and Wayne assay performance analysis in a tuberculosis cohort in Lima, Peru. Int J Tuberc Lung Dis 21:894–901. doi:10.5588/ijtld.16.0850. 28786798 PMC5555119

[45] Caviedes L, Lee T-S, Gilman RH, Sheen P, Spellman E, Lee EH, Berg DE, Montenegro-James S, Tuberculosis Working Group in Peru. 2000. Rapid, efficient detection and drug susceptibility testing of Mycobacterium tuberculosis in sputum by microscopic observation of broth cultures. J Clin Microbiol 38:1203–1208. doi:10.1128/JCM.38.3.1203-1208.2000. 10699023 PMC86377

[46] World Health Organization. 2021. Catalogue of mutations in Mycobacterium tuberculosis complex and their association with drug resistance. World Health Organization, Geneva, Switzerland. https://www.who.int/publications/i/item/9789240028173.

[47] CRyPTIC Consortium and the 100,000 Genomes Project, Allix-Béguec C, Arandjelovic I, Bi L, Beckert P, Bonnet M, Bradley P, Cabibbe AM, Cancino-Muñoz I, Caulfield MJ, Chaiprasert A, Cirillo DM, Clifton DA, Comas I, Crook DW, De Filippo MR, de Neeling H, Diel R, Drobniewski FA, Faksri K, Farhat MR, Fleming J, Fowler P, Fowler TA, Gao Q, Gardy J, Gascoyne-Binzi D, Gibertoni-Cruz A-L, Gil-Brusola A, Golubchik T, Gonzalo X, Grandjean L, He G, Guthrie JL, Hoosdally S, Hunt M, Iqbal Z, Ismail N, Johnston J, Khanzada FM, Khor CC, Kohl TA, Kong C, Lipworth S, Liu Q, Maphalala G, Martinez E, Mathys V, Merker M, Miotto P, et al. 2018. Prediction of susceptibility to first-line tuberculosis drugs by DNA sequencing. N Engl J Med 379:1403–1415. doi:10.1056/NEJMoa1800474. 30280646 PMC6121966

[48] Chernyaeva EN, Shulgina MV, Rotkevich MS, Dobrynin PV, Simonov SA, Shitikov EA, Ischenko DS, Karpova IY, Kostryukova ES, Ilina EN, Govorun VM, Zhuravlev VY, Manicheva OA, Yablonsky PK, Isaeva YD, Nosova EY, Mokrousov IV, Vyazovaya AA, Narvskaya OV, Lapidus AL, O’Brien SJ. 2014. Genome-wide Mycobacterium tuberculosis variation (GMTV) database: a new tool for integrating sequence variations and epidemiology. BMC Genomics 15:308. doi:10.1186/1471-2164-15-308. 24767249 PMC4234438

[49] Sandgren A, Strong M, Muthukrishnan P, Weiner BK, Church GM, Murray MB. 2009. Tuberculosis drug resistance mutation database. PLoS Med 6:e2. doi:10.1371/journal.pmed.1000002. 19209951 PMC2637921

[50] Karmakar M, Rodrigues CHM, Horan K, Denholm JT, Ascher DB. 2020. Structure guided prediction of pyrazinamide resistance mutations in pncA. Sci Rep 10:1875. doi:10.1038/s41598-020-58635-x. 32024884 PMC7002382

[51] Velásquez GE, Calderon RI, Mitnick CD, Becerra MC, Huang C-C, Zhang Z, Contreras CC, Yataco RM, Galea JT, Lecca LW, Murray MB. 2016. Pyrazinamide resistance assays and two-month sputum culture status in patients with multidrug-resistant tuberculosis. Antimicrob Agents Chemother 60:6766–6773. doi:10.1128/AAC.00632-16. 27600032 PMC5075084

