# Supplementary material for: Quantitative 1H Nuclear Magnetic Resonance Assay for the Rapid Detection of Pyrazinamide Resistance in Mycobacterium tuberculosis from Sputum Samples
Source: J Clin Microbiol. 2023 Apr 18;61(5):e01522-22. doi: 10.1128/jcm.01522-22 (PMC10204627; doi:10.1128/jcm.01522-22)
Supplement: Supplemental file 1 — Supplemental material. Download jcm.01522-22-s0001.pdf, PDF file, 0.7 MB [file jcm.01522-22-s0001.pdf]

Supporting Data:

Quantitative ^{1}H-NMR assay from sputum samples for a rapid detection of pyrazinamide resistance in *Mycobacterium tuberculosis*

Juan M. Lopez[1*], Mirko Zimic[2], Katherine Vallejos[2], Diego Sevilla[1], Mariella Quispe-Carbajal[1], Elisa Roncal[2], Joseline Rodríguez[2], Jhojailith Rodríguez[2], Ricardo Antiparra[2], Héctor Arteaga[2], Robert H. Gilman[3], Helena Maruenda[1*], Patricia Sheen[2*].

[1] Departamento de Ciencias – Química, CERMN, Pontificia Universidad Católica del Perú, Av. Universitaria 1801, Lima 32, Perú.

[2] Laboratorios de Investigación y Desarrollo, Facultad de Ciencias y Filosofía, Universidad Peruana Cayetano Heredia, Av. H. Delgado 430, SMP, Lima 31, Perú.

[3] Johns Hopkins Bloomberg School of Public Health, Baltimore, MD 21205, United States

*Corresponding authors: juan.lopez@pucp.edu.pe, hmaruen@pucp.edu.pe, and patricia.sheen@upch.pe

Supporting Figures:

Index 0		Index 1		Index 2		Index 3		Index 4	
Control	PZA	Control	PZA	Control	PZA	PZA	Control	Control	PZA

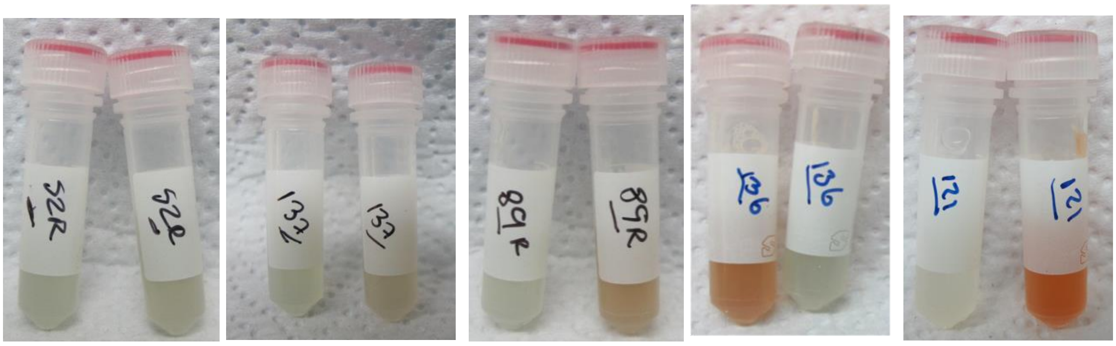

Figure S1. Representative color Index in MODS-Wayne assays. Left, control sample without SAF; right sample with SAF. Color intensities are classified as absence of color (Index 0), low (Index 1), mid (Index 2), high (Index 3), and extreme (Index 4).

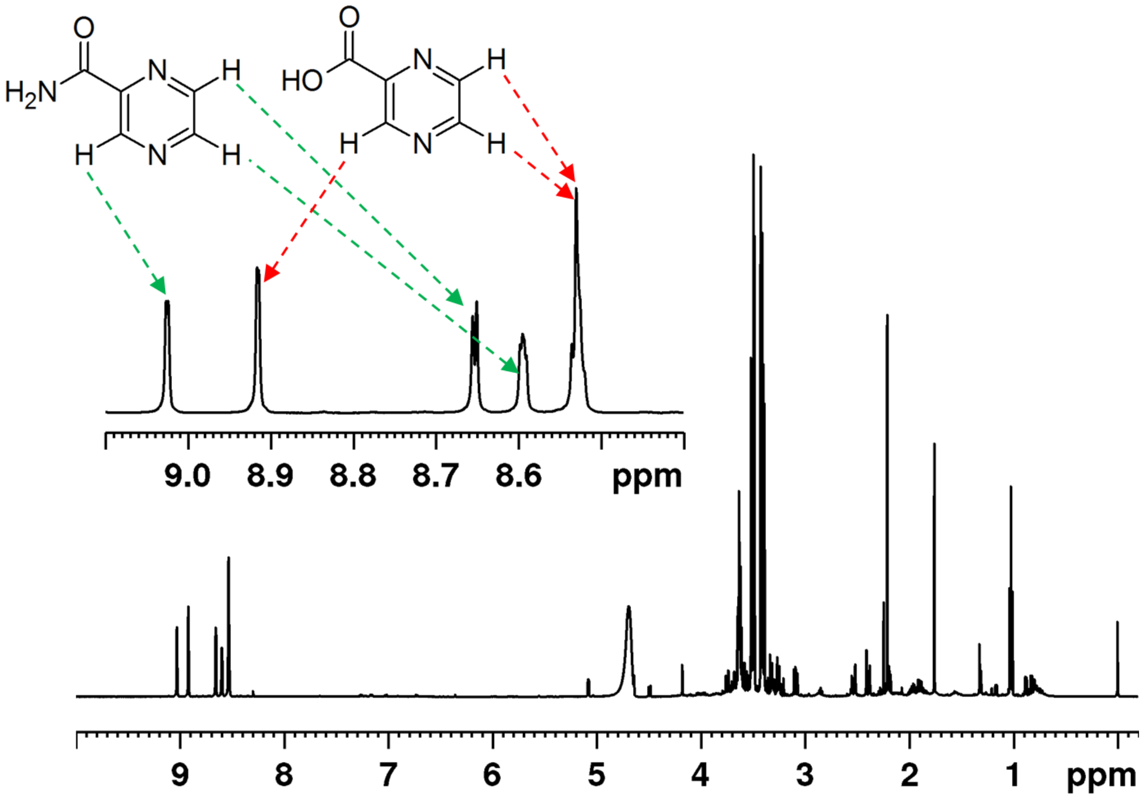

Figure S2. ^{1}H-NMR spectrum of *Mtb*-positive MODS culture supernatant (Sample TBCA-209) after three days of incubation with PZA. Upper left, a magnification of the area between 8.5 and 9.1 ppm with the assignment of the aromatic signals for PZA (9.02 ppm, 8.65 ppm, and 8.59 ppm) and POA (8.91 ppm and 8.53 ppm).

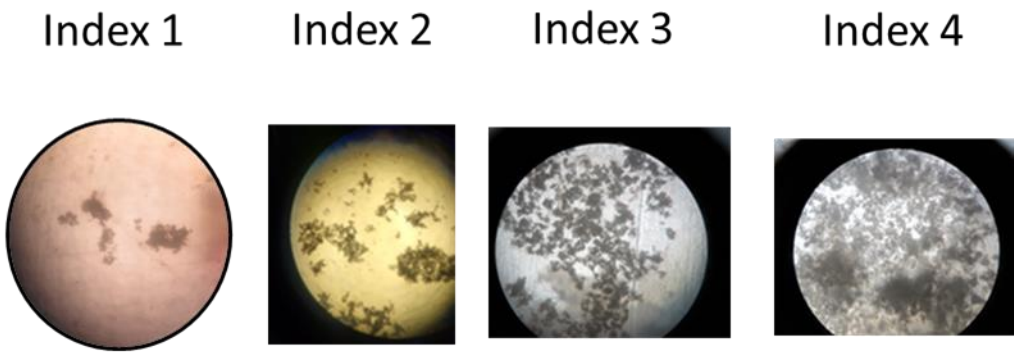

Figure S3. Representative results of cordon-pattern growth observation and classification according to MODS Growth Index. Index 1, growth covering 25% of the well area; Index 2, growth covering 25 – 50% of the well area; Index 3: growth covering 50 – 75% of the well area; and Index 4, growth covering 100% of the well area.

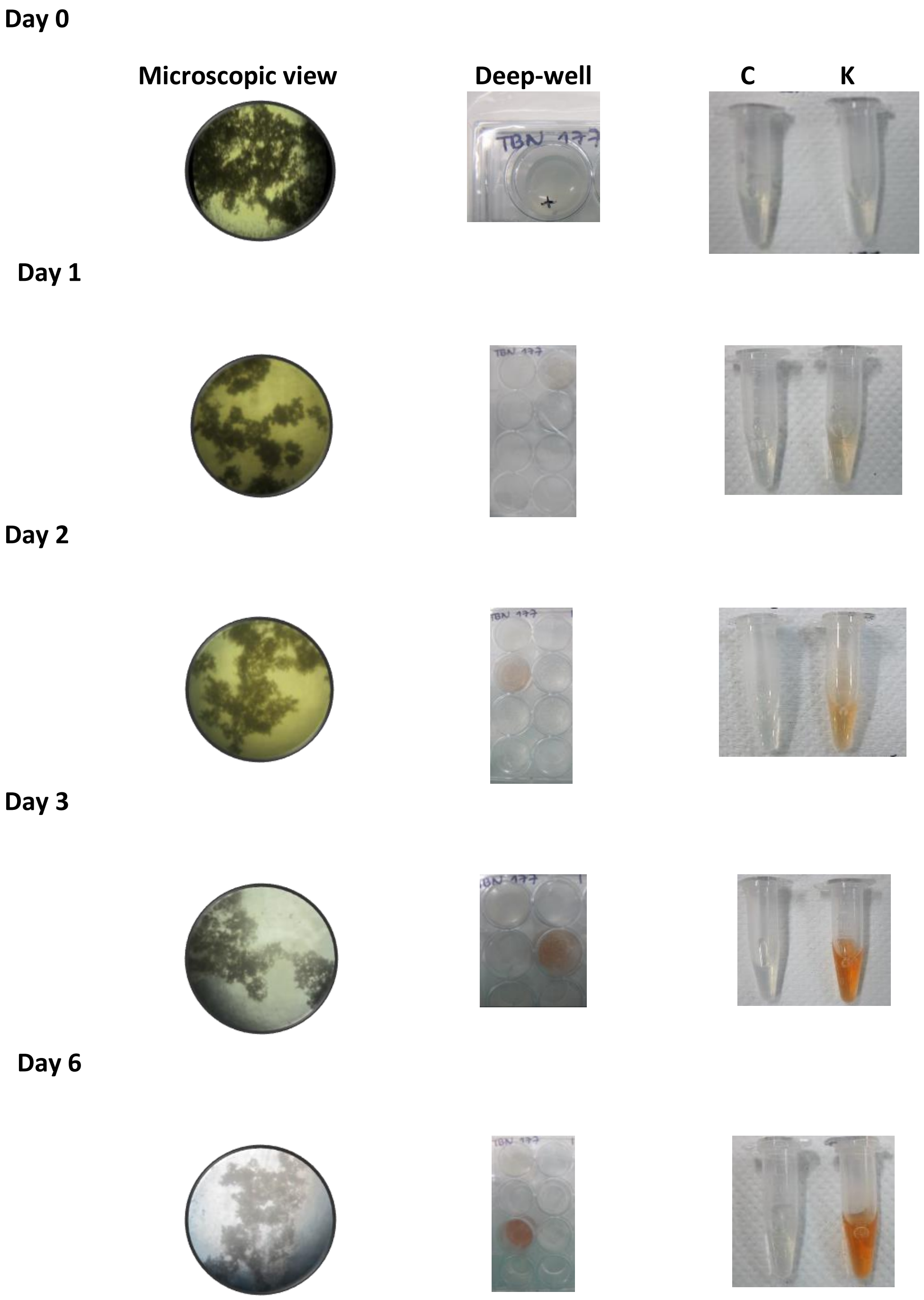

Figure S4. The kinetics of the sample TBN-177 monitored by the MODS-WAYNE semi-quantitative assay. C: Control sample without SAF; K: Sample with SAF added.

Supporting Tables:

	Drug Sensibility Test		
	**Wayne**	**MGIT**	***pnc*A-Seq**
**Resistant**	34/139 (24.5%)	49/139 (35.3%)	43/139 (30.9%)
**Susceptible**	105/139 (75.5%)	90/139 (64.7%)	96/139 (69.1%)

Table S1. Susceptibility to PZA determined by regular Wayne, MGIT, and *pnc*A-Seq.

GENOTYPE	FREQUENCY	PZA PROFILE	PERCENTAGE
407insA/461insA	1	RESISTANT	0.7 %
D49N	2	RESISTANT	1.4 %
D8E	1	RESISTANT	0.7 %
F81S	4	RESISTANT*	2.9 %
G124A	2	SUSCEPTIBLE	1.4 %
H51R	12	RESISTANT	8.6 %
H57L	3	RESISTANT	2.2 %
H82D	1	RESISTANT	0.7 %
L4S	1	RESISTANT	0.7 %
Promotor A-11G	1	RESISTANT	0.7 %
Q10R	11	RESISTANT	7.9 %
T160P	1	RESISTANT	0.7 %
V180F	3	RESISTANT	2.2 %
V9G	1	RESISTANT	0.7 %
Δ456-466	1	RESISTANT	0.7 %
Wild-type	94	SUSCEPTIBLE	67.6 %

Table S2. Mutations of promotor and *pnc*A gene found in this study. Specific mutations are reported along with the strain's corresponding PZA susceptibility profile according to "Catalogue of mutations in *Mycobacterium tuberculosis* complex and their association with drug resistance"[1].* F81S mutation is described as susceptible in the WHO database while several recent articles report it as a resistant strain[2–6].

DRUG SUSCEPTIBLE TEST			Number of isolates	Percentage
**Wayne**	**MGIT**	***pnc*A-Seq**		
Susceptible	Susceptible	Susceptible	82/139	59.0 %
Resistant	Resistant	Resistant	32/139	23.0 %
Susceptible	Resistant	Susceptible	12/139	8.6 %
Susceptible	Susceptible	Resistant	6/139	4.3 %
Susceptible	Resistant	Resistant	5/139	3.6 %
Resistant	Susceptible	Susceptible	2/139	1.4 %
Resistant	Resistant	Susceptible	0/139	0 %
Resistant	Susceptible	Resistant	0/139	0 %
**CRS**				
	**RESISTANT**		37/139	26.6 %
	**SUSCEPTIBLE**		102/139	73.4 %

Table S3. Sample distribution by PZA susceptibility profile according to Wayne, MGIT, *pncA-Seq,* and CRS. R: Resistant, S: Susceptible, CRS: Composite Reference Standard.

	Drug Susceptibility Test							
	Wayne		MGIT		*pnc*A-Seq		CRS	
	S	R	S	R	S	R	S	R
**Average**	29.5%	5.6%	28.4%	15.0%	31.7%	5.7%	30.4%	4.4%
**Standard deviation**	24.5%	6.6%	23.4%	23.2%	24.5%	9.5%	24.7%	4.5%
**Median**	21.7%	2.4%	20.4%	5.9%	24.0%	2.4%	23.1%	2.4%
**First Quartile**	11.2%	1.2%	12.0%	1.3%	13.4%	1.1%	12.3%	1.1%
**Third Quartile**	42.1%	8.3%	35.6%	12.8%	43.1%	7.9%	42.2%	7.0%
**Interquartile Range**	30.9%	7.1%	23.6%	11.5%	29.7%	6.8%	29.9%	5.9%
P value	$1.8*10^{-7}$		$1.7*10^{-3}$		$5.4*10^{-10}$		$5.6*10^{-9}$	

Table S4. Statistical parameters of % POA values distribution according to PZA susceptibility determined by Wayne, MGIT, *pncA-Seq*, and CRS. R: Resistant, S: Susceptible, CRS: Composite Reference Standard. P value obtained from Student's T-test.

MODS-NMR				
**Cut off**	**Test**	**Sensitivity**	**Specificity**	**Kappa Index**
**7.4 % POA**	**CRS**	91,20%	78,40%	0,69
	***pnc*A-seq**	93,80%	74,40%	0,704
	**Wayne**	86,70%	70,60%	0,551
	**MGIT**	91,10%	61,20%	0,552
**9.4 % POA**	**CRS**	84,30%	94,60%	0,704
	***pnc*A-seq**	86,50%	88,40%	0,716
	**Wayne**	80,00%	88,20%	0,584
	**MGIT**	83,30%	73,50%	0,563
**20.6 % POA**	**CRS**	52,00%	100,00%	0,365
	***pnc*A-seq**	54,20%	97,70%	0,406
	**Wayne**	49,50%	97,10%	0,37
	**MGIT**	47,80%	79,60%	0,234
**MODS-Wayne**				
**Index 1**	**CRS**	92,00%	80,00%	0,713
	***pnc*A-seq**	94,70%	75,60%	0,728
	**Wayne**	88,40%	75,00%	0,607
	**MGIT**	92,10%	61,70%	0,569

Table S5. Sensitivity, specificity, and Kappa Index reported for the MODS-NMR assay at three different thresholds (7.4% POA, 9.4% POA and 20.6% POA) and for the MODS-Wayne assay at Index 1 threshold.

	**BK**			
	**0**	**1**	**2**	**3**
**Average**	27.4%	35.6%	42.2%	37.6%
**Standard deviation**	22.2%	26.8%	30.8%	26.6%
**Median**	23.5%	28.2%	30.0%	36.5%
**First Quartile**	7.4%	15.6%	18.0%	14.6%
**Third Quartile**	51.3%	56.6%	62.6%	56.1%
**Interquartile Range**	43.9%	41.0%	44.5%	41.5%
**ANOVA p-value**	0.68			

Table S6. Statistical parameters of % POA values distribution according to BK values. Only data from patients diagnosed as positive by CRS were used in this analysis. One-way ANOVA p-value is also reported.

	MODS Growth Index			
	1	2	3	4
**Average**	21.3%	34.1%	34.1%	40.0%
**Standard deviation**	22.3%	25.2%	25.8%	29.1%
**Median**	13.1%	25.3%	32.4%	34.3%
**First Quartile**	9.0%	17.6%	8.5%	15.3%
**Third Quartile**	25.4%	42.5%	54.1%	66.6%
**Interquartile Range**	16.4%	24.9%	45.6%	51.3%
**ANOVA P value***	0.099			
**ANOVA P value****	$3.9*10^{-4}$			

Table S7. Statistical parameters of % POA values distribution according to MODS-GI values. Only data from patients diagnosed as positive by CRS were used in this analysis. One-way ANOVA test was performed with* and without** statistical outliers; p-values are reported in the table.

	MODS Growth Index	
	Tukey HSD p-value	Tukey HSD inference
**Index 1 vs Index 2**	0.011	p<0.05
**Index 1 vs Index 3**	0.003	p<0.01
**Index 1 vs Index 4**	0.006	p<0.01
**Index 2 vs Index 3**	0.894	insignificant
**Index 2 vs Index 4**	0.564	insignificant
**Index 3 vs Index 4**	0.869	insignificant

Table S8. Post-hoc Tukey HSD test was performed on % POA distribution according to MODS-GI after removing statistical outliers (see Figure 6 and Table S7). Statistical significant difference between groups: p-value p>0.05 statistically insignificant; p<0.05 statistically significant; p<0.01 statistically highly significant.

	MODS-WAYNE INDEX				
	**0**	**1**	**2**	**3**	**4**
**Average**	3.2%	14.4%	26.1%	54.4%	72.5%
**Standard deviation**	4.3%	9.6%	12.8%	18.9%	24.2%
**Median**	1.7%	12.4%	23.7%	56.6%	77.6%
**First Quartile**	1.0%	8.4%	17.2%	43.2%	53.2%
**Third Quartile**	3.6%	17.4%	30.0%	68.2%	97.2%
**Interquartile Range**	2.5%	8.9%	12.8%	25.0%	44.0%

Table S9. Statistical parameters of % POA values distribution according to MODS-WI values.

Patient ID	Sample ID	Sampling day	BK	MODS Positive Day	MODS Growth Index	Wayne Test	MGIT	Promotor *pnc*A - Sequencing	CRS	MODS-Wayne	MODS-Wayne INDEX	POA (%)	INH R	RIF R
TB_002	TBN-45	0	++	5	1	R	R	V180F	R	S	2	8,9	R	R
	TBN-56	7	ND	20	1	R	R	V180F	R	R	0	0,0	R	R
	TBN-77	14	ND	22	2	S	S	V180F	S	R	0	0,0	R	R
TB_008	TBN-120	0	+++	6	1	S	S	WT	S	S	1	7,1	R	S
	TBN-154	14	ND	10	1	S	S	WT	S	S	1	13,5	R	S
TB_009	TBN-131	0	+++	7	3	S	R	F81S	R	S	1	1,1	R	R
	TBN-153	7	ND	10	3	S	S	F81S	S	S	1	1,9	R	R
	TBN-166	14	ND	9	3	S	S	F81S	S	R	0	3,6	R	R
TB_012	TBN-148	0	+	6	2	S	S	WT	S	S	2	24,2	R	R
	TBN-214	7	ND	13	1	S	S	WT	S	S	1	11,5	R	R
TB_014	TBN-170	7	ND	14	1	R	S	WT	S	S	1	19,8	R	S
	TBN-184	14	ND	17	1	S	S	WT	S	S	1	8,8	S	S
TB_015	TBN-176	7	ND	17	2	S	S	WT	S	S	2	17,9	S	S
	TBN-186	14	ND	17	2	S	S	WT	S	S	2	16,7	S	S
TB_016	TBN-177	0	+++	17	3	S	S	WT	S	S	3	71,5	S	S
	TBN-194	14	ND	17	1	S	S	WT	S	S	1	7,5	S	S
TB_017	TBN-182	0	+	17	2	S	S	WT	S	S	2	21,0	S	S
	TBN-192	7	ND	22	2	S	S	WT	S	S	2	23,1	S	S
	TBN-199	14	ND	31	1	S	S	WT	S	S	1	10,1	S	S
TB_018	TBN-187	0	++	14	3	S	S	WT	S	S	3	55,8	S	S
	TBN-204	7	ND	15	1	S	S	WT	S	S	1	14,8	S	S
	TBN-215	14	ND	29	1	S	S	WT	S	S	1	17,1	S	S

Patient ID	Sample ID	Sampling day	BK	MODS Positive Day	MODS Growth Index	Wayne Test	MGIT	Promotor *pnc*A - Sequencing	CRS	MODS-WAYNE	MODS-WAYNE INDEX	POA (%)	INH R	RIF R
TB_018	TBN-187	0	++	14	3	S	S	WT	S	S	3	55,8	S	S
	TBN-204	7	ND	15	1	S	S	WT	S	S	1	14,8	S	S
	TBN-215	14	ND	29	1	S	S	WT	S	S	1	17,1	S	S
TB_023	TBN-193	0	+++	6	2	S	S	WT	S	S	2	13,8	R	R
	TBN-201	7	ND	8	1	S	S	WT	S	S	1	13,8	R	R
	TBN-213	14	ND	10	1	S	S	WT	S	S	1	17,7	R	R
TB_027	TBN-206	0	++	13	1	S	S	WT	S	S	1	12,9	S	S
	TBN-218	7	ND	13	2	S	S	WT	S	S	2	9,5	S	S
TB_028	TBN-211	0	-	10	3	S	S	WT	S	S	3	51,3	S	S
	TBN-219	7	ND	13	3	S	S	WT	S	S	3	50,2	S	S
TB_029	TBN-217	7	ND	13	1	S	S	WT	S	S	1	12,1	S	S
	TBN-222	14	ND	15	2	S	S	WT	S	S	2	35,2	S	S
TB_032	TBN-230	0	+	14	2	S	S	WT	S	S	2	27,9	S	S
	TBN-236	14	ND	30	1	S	S	WT	S	S	1	13,3	S	S
TB_033	TBN-231	0	+	21	1	S	S	WT	S	S	1	8,1	S	S
	TBN-233	7	ND	16	1	S	S	WT	S	S	1	32,6	S	S
TB_034	TBN-262	0	+	11	3	R	R	H57L	R	R	0	1,2	R	R
	TBN-269	14	ND	10	3	R	R	H57L	R	R	0	1,6	R	R
TB_035	TBN-297	0	ND	8	1	S	S	WT	S	S	1	1,2	R	R
	TBN-304	7	ND	8	1	S	S	F81S	S	S	1	9,4	R	R
	TBN-324	14	ND	10	2	S	S	WT	S	S	2	26,3	R	R

Table S10: Full data for 17 TB-patients at each sampling day (0, 7, and 14). R= Resistant. S = Susceptible. BK= baciloscopy. INH R = Isoniazid Resistant. RIF R= Rifampicin Resistant. ND = No Data.

**Kappa Index**	***pnc*A-seq**	**MGIT**	**MODS-NMR**	**MODS-Wayne**
**Wayne**	0.768	0.678	0.584	0.607
***pnc*A-seq**		0.708	0.716	0.728
**MGIT**			0.563	0.569
**MODS-NMR**				0.711

Table S11: Kappa coefficient of Agreement between drug susceptibility assays.

Patient ID	Sample ID	Sampling day	BK	MODS Positive Day	MODS Growth Index	Wayne Test	MGIT	Promotor - *pnc*A Sequencing	CRS	MODS-Wayne	MODS-Wayne Index	POA (%)	INH	RIF
TB_082	TBCA-174	0	++	10	2	S	R	Q10R	R	R	0	6,3	R	R
TB_099	TBCA-214	0	+++	12	3	S	R	Q10R	R	R	0	4,3	R	R
TB_107	TBCA-240	0	ND	7	3	S	R	H51R	R	R	0	1,0	R	R
TB_113	TBCA-250	0	ND	7	4	S	R	H51R	R	R	0	7,2	R	R
TB_002	TBN-77	14	ND	22	2	S	S	V180F	S	R	0	0,0	R	R
TB_009	TBN-166	14	ND	9	3	S	S	F81S	S	R	0	3,6	R	R
TB_067	TBCA-94	0	+++	6	4	S	R	WT	S	R	0	1,3	R	R
TB_003	TBN-52	7	ND	17	1	S	S	WT	S	R	0	0,0	R	R
TB_078	TBCA-167	0	+++	8	4	S	S	WT	S	R	0	2,0	R	R
TB_089	TBCA-186	0	+	21	1	S	S	WT	S	R	0	3,5	R	R
TB_096	TBCA-211	0	+	12	3	S	S	WT	S	R	0	8,9	R	R
TB_001	ME-27	0	ND	ND	3	R	R	D49N	R	S	3	0,9	R	R
TB_002	TBN-45	0	++	5	1	R	R	V180F	R	S	2	8,9	R	R
TB_048	MP-465	0	+	16	1	R	R	Prom A-11G	R	S	ND	6,0	R	S
TB_050	MP-505	0		ND	4	R	R	D49N	R	S	ND	6,0	R	R
TB_074	TBCA-150	0	+++	7	2	R	R	L4S	R	S	1	9,4	R	R
TB_009	TBN-131	0	+++	7	3	S	R	F81S	R	S	1	1,1	R	R
TB_009	TBN-153	7	ND	10	3	S	S	F81S	S	S	1	1,9	R	R
TB_035	TBN-304	7	ND	8	1	S	S	F81S	S	S	1	9,4	R	R

Patient ID	Sample ID	Sampling day	BK	MODS Positive Day	MODS Growth Index	Wayne Test	MGIT	Promotor - *pnc*A Sequencing	CRS	MODS-Wayne	MODS-Wayne Index	POA (%)	INH	RIF
TB_007	TBN-90	0	ND	32	1	S	S	WT	S	S	1	7,9	S	S
TB_008	TBN-120	0	+++	6	1	S	S	WT	S	S	1	7,1	R	S
TB_016	TBN-194	14	ND	17	1	S	S	WT	S	S	1	7,5	S	S
TB_019	TBN-188	0	-	17	1	S	S	WT	S	S	1	7,4	S	S
TB_025	TBN-202	0	++	8	3	S	S	WT	S	ND	ND	7,5	S	S
TB_033	TBN-231	0	+	21	1	S	S	WT	S	S	1	8,1	S	S
TB_035	TBN-297	0	ND	8	1	S	S	WT	S	S	1	1,2	R	R
TB_079	TBCA-171	0	+	18	1	S	S	V9G	S	R	0	10,0	R	R
TB_100	TBCA-215	0	+++	12	2	R	R	Q10R	R	S	1	13,3	R	S
TB_014	TBN-170	7	ND	14	1	R	S	WT	S	S	1	19,8	R	S
TB_051	TBCA-2	0	+	17	ND	R	S	WT	S	S	2	28,6	R	R
TB_059	TBCA-43	0	+	16	2	S	R	WT	S	S	3	56,9	R	R
TB_063	TBCA-68	0	+	10	1	S	R	WT	S	S	4	100,0	R	S
TB_064	TBCA-79	0	+	14	1	S	R	WT	S	S	3	40,9	R	S
TB_065	TBCA-82	0	++	7	2	S	R	WT	S	S	2	37,3	R	S
TB_066	TBCA-87	0	+++	7	3	S	R	WT	S	S	4	53,6	R	R
TB_070	TBCA-111	0	+++	7	3	S	R	G124A	S	S	2	45,8	R	S
TB_071	TBCA-115	0	+	8	2	S	R	WT	S	S	3	56,2	R	S
TB_072	TBCA-137	0	+++	10	2	S	R	WT	S	S	4	89,9	R	S
TB_073	TBCA-141	0	+	18	1	S	R	WT	S	S	1	12,3	R	R
TB_090	TBCA-187	0	++	19	2	S	R	WT	S	S	2	43,4	R	R
TB_091	TBCA-191	0	+++	17	1	S	R	WT	S	S	3	36,5	R	R

Table S12: Full data for the 39 samples that contain at least one discordant result. R= Resistant. S = Susceptible. BK= baciloscopy. INH R = Isoniazid Resistant. RIF R= Rifampicin Resistant. ND = No Data .

**Wayne**	S	S	S	S	R	S	S	S	S	R	R	S
pncA-seq	R	R	S	S	R	R	R	S	R	R	S	S
**MGIT**	R	S	R	S	R	R	S	S	S	R	S	R
**MODS-NMR**	R	R	R	R	R	R	R	R	S	S	S	S
**MODS-Wayne**	R	R	R	R	S	R	S	S	R	S	S	S
**TOTAL**	4	2	1	4	5	1	2	7	1	1	2	11

Table S13: Summary of the disagreement observed among the 39 samples with respect to the DST employed. R= Resistant, S = Susceptible.

	**Wayne**	***pncA-seq***	**MGIT**	**MODS-NMR**	**MODS-Wayne**
**Wayne**		12	19	24	21
pncA-seq	8,6%		17	14	13
**MGIT**	13,7%	12,2%		27	24
**MODS-NMR**	17,3%	10,1%	19,4%		15
**MODS-Wayne**	15,1%	9,4%	17,2%	10,8%	

Table S14: Discrepancies between each drug susceptibility test: top, number of discrepancies and bottom, percentage of discrepancy.

References

1. World Health Organization. *Catalogue of mutations in Mycobacterium tuberculosis complex and their association with drug resistance*. (2021).
2. Allix-Béguec, C. *et al.* Prediction of Susceptibility to First-Line Tuberculosis Drugs by DNA Sequencing. *N. Engl. J. Med.* **379**, 1403–1415 (2018).
3. Chernyaeva, E. N. *et al.* Genome-wide Mycobacterium tuberculosis variation (GMTV) database: A new tool for integrating sequence variations and epidemiology. *BMC Genomics* **15**, 1–8 (2014).
4. Sandgren, A. *et al.* Tuberculosis Drug Resistance Mutation Database. *PLOS Med.* **6**, (2009).
5. Yadon, A. N. *et al.* A comprehensive characterization of *PncA* polymorphisms that confer resistance to pyrazinamide. *Nat. Commun.* **8**, 1–10 (2017).
6. Karmakar, M., Rodrigues, C. H. M., Horan, K., Denholm, J. T. & Ascher, D. B. Structure guided prediction of Pyrazinamide resistance mutations in *pncA*. *Sci. Rep.* **10**, 1–10 (2020).
